# IGFBP-6 regulates SH2D4A expression to promote breast cancer cell cycle progression in response to progesterone

**DOI:** 10.3389/fendo.2026.1856096

**Published:** 2026-06-30

**Authors:** Francisco J. Lariz, Diana C. Bautista-Tovar, Alejandro Lazo-Loya, Kevin D. Houston

**Affiliations:** New Mexico State University, Las Cruces, NM, United States

**Keywords:** breast cancer, cell cycle, IGFBP-6, progesterone receptor, SH2D4A

## Abstract

Insulin-like growth factor binding protein-6 (IGFBP-6) is induced by progesterone in breast cancer cells and regulates progesterone receptor (PR) via negative feedback. To further understand the mechanisms by which IGFBP-6 regulates PR and downstream signaling, proteomic analysis was performed in progesterone-treated T47D breast cancer cells following siRNA-mediated knockdown of IGFBP-6. Of the 8638 unique proteins identified, 29 proteins were downregulated, and 14 proteins were upregulated by progesterone treatment after IGFBP-6 knockdown. The decreased proteins were mostly identified as regulators of G2/M phase of the cell cycle. SH2D4A is induced by progesterone only when IGFBP-6 was high. To understand the role of SH2D4A in progesterone-induced signaling, SH2D4A was knocked down using siRNA prior to progesterone treatment. Knockdown of IGFBP-6 or SH2D4A leads to cell cycle arrest in G1, and both SH2D4A and IGFBP-6 regulate cyclin B. Treatment of cells with abemaciclib and nocodazole caused cell cycle arrest in G1 and G2/M, respectively, and decreased IGFBP-6, suggesting that IGFBP-6 is regulated in a cell cycle-dependent manner. These results identify a pathway linking progesterone to IGFBP-6 and SH2D4A, and to cell cycle progression in breast cancer cells.

## Introduction

Breast cancer is the second most common cancer worldwide, accounting for 2.3 million new cases and over 665,000 deaths in 2022 (1, 2). Breast cancers are classified based on the expression of Estrogen receptor (ER), Progesterone Receptor (PR), and Human Epidermal Growth Factor Receptor 2 (HER2) to guide therapy and predict patient outcomes (3). In ER and PR positive breast cancer, high levels of Insulin-like growth factor binding protein-6 (IGFBP-6) result in improved outcomes for breast cancer patients (4–6). IGFBP-6 is one of the IGFBPs that sequester Insulin-like growth factors (IGFs) to prolong their half-life and attenuate binding to Insulin-like growth factor 1 receptor (7–10). In breast cancer, IGFBP-6 is described as having a tumor-suppressive role, as IGFBP-6 levels are generally lower in malignant tissue than in healthy tissue (4, 11).

IGFBP-6 is induced by progesterone in breast cancer cells and in the rat myometrium (5, 12). Progesterone (P4) is an endogenous steroid hormone that has roles in puberty and pregnancy (13, 14). In breast cancer, progesterone has multiple roles, including antagonism of other steroid hormone activity and regulation of cell cycle progression (15–17). IGFBP-6 is a downstream regulator of progesterone signaling and is required for the antagonism of estradiol-stimulated proliferation (5). Knockdown of IGFBP-6 destabilizes PR, prevents induction of p21 (Cip/Waf), and promotes expression of cyclin E2 (5). Both progesterone and IGFBP-6 regulate cell cycle progression. Progesterone regulates the cell cycle in a dual manner by inducing the cell cycle inhibitors p21 and p27 (16) while also being able to activate mitogenic signaling and induction of cyclin D1 and cyclin E (17). IGFBP-6 also plays a role in cell cycle progression, regulating exit from G1 in breast cancer cells (18). IGFBP-6 knockdown decreases the expression of cyclin B1 and the percentage of cells in G2/M (18). This is similar to the effects of inhibiting PR, which decreases the expression of proteins associated with G2/M and prevents the induction of IGFBP-6 (5, 19). These studies suggest that IGFBP-6 acts downstream to PR as a regulator of the cell cycle.

This study investigated the effects of progesterone and IGFBP-6 on the cell cycle in breast cancer cells. A proteomics analysis was done to better understand the consequences of IGFBP-6 knockdown in progesterone signaling. In total, 8638 unique proteins were identified. When IGFBP-6-low cells were treated with progesterone, 29 proteins were downregulated, and 14 were upregulated. Among the decreased proteins was SH2D4A, whose induction by progesterone was blocked by IGFBP-6 knockdown. Survival analysis showed that SH2D4A confers improved overall, recurrence-free, and distant metastasis-free survival in luminal A breast cancers. Knockdown of both IGFBP-6 and SH2D4A resulted in accumulation of cells in G1, and a decrease of cells in G2/M. Knockdown of SH2D4A also resulted in lower accumulation of cyclin B1 and p21. Finally, cell cycle arrest at G1 and G2 demonstrates that intracellular IGFBP-6 levels exhibit cell cycle phase-regulated expression. These results help identify a novel pathway linking progesterone, IGFBP-6, and SH2D4A to cell cycle progression.

## Materials and methods

Many of these methods were described previously (5, 18, 20).

### Survival analysis

Kaplan-Meier survival plots were produced from data in the KM Plotter database, which uses RNA-seq expression and Genechip microarray data obtained from the European Genome-Phenome Archive (EGA) and the Gene Expression Omnibus (GEO) (21). Patients were divided by median expression of SH2D4A in patients with luminal A breast cancer, defined by the St. Gallen Classification (3).

### Cell culture

T47D, MCF-7, MDA-MB-231, and MDA-MB-468 breast cancer cells were acquired from ATCC (Manassas, VA). T47D-Y cells were a generous gift from Dr. Carol Lange (University of Minnesota). Cells were maintained in DMEM supplemented with 10% fetal bovine serum, 1mM sodium pyruvate, and 2mM L-glutamine (Life Technologies, Carlsbad, CA). Cells below passage 15 were used except for T47D Y cells, which were at a passage between 45 and 50, and all RNA and protein purifications were performed on cells at a similar confluence.

### Steroid hormone and antiprogestin treatments

Cells were treated with 10nM estradiol (E2) (Millipore Sigma, St. Louis, MO), 50nM progesterone (P4) (Millipore Sigma, St. Louis, MO), or 10nM E2 plus 50nM P4 in ethanol. Cells were also treated with 25nM Mifepristone (RU 486) (Millipore Sigma, St. Louis, MO) either alone or in the presence of 50nM P4. Cells were plated and after 24 hours, washed with 1X PBS and then treated in DMEM supplemented with 10% Charcoal-stripped FBS, 1mM sodium pyruvate, and 2mM L-glutamine (Life Technologies, Carlsbad, CA) plus the corresponding steroid or antiprogestin treatment.

### Nocodazole and abemaciclib treatments

Cells were treated with 0.1% DMSO, 100nM, 1μM, or 10 μM doses of nocodazole (Millipore Sigma, St. Louis, MO). Cells were treated with 0.1% DMSO, 5nM, 50nM or 500nM doses of abemaciclib (MedChem Express, Monmouth Junction, NJ).

### Immunoblot analysis

Cells were lysed with RIPA buffer containing protease and phosphatase inhibitor cocktails (Life Technologies, Carlsbad, CA). After lysis, the cells were centrifuged at 9,000 x g for 20min at 4 °C, and the supernatant was collected. Protein concentrations were determined by BCA assay (Thermo Scientific, Rockford, IL). 10-30μg of protein was resolved using Bolt 4-12% Bis-Tris Plus gels and dry transferred to a PVDF membrane with the iBlot2 system (Life Technologies, Carlsbad, CA). Membranes were blocked in 1X Tris-buffered saline 0.1% Tween 20 (TBST) and either 10% Bovine Serum Albumin (BSA) (Thermo Scientific, Rockford, IL) for phospho-proteins or 5% fat-free milk for all other protein targets. Membranes were then washed in 1X TBST three times, and the primary antibody was added and incubated overnight at 4 °C. Proteins were incubated at a ratio of 1:1000 in 5% BSA in TBST for phosphoproteins or in 5% milk in TBST for other proteins. Antibodies used are listed as follows: IGFBP-6 (#ab219560, Abcam, Waltham, MA), Cyclin B1 (#12231 Cell Signaling Technology, Danvers, MA), SH2D4A (#PA5-51648, Thermo Scientific, Rockford, IL), PR (#8757 Cell Signaling Technology, Danvers, MA), p21(Cip/Waf) (#2947 Cell Signaling Technology, Danvers, MA), ER Alpha (#8644 Cell Signaling Technology, Danvers, MA), and Beta Actin (#sc 47778, Santa Cruz Biotechnology, Dallas, TX).

After incubation with primary antibodies, membranes were washed three times with 1X TBST then incubated with either anti-rabbit IgG conjugated to horseradish peroxidase (#7074, Cell Signaling Technology, Danvers, MA) or anti-mouse IgG conjugated to horseradish peroxidase (#7076, Cell Signaling Technology, Danvers, MA) for 1hr at room temperature with a dilution ratio of 1:5000. Membranes were then washed three times with 1X TBST before Supersignal ™ chemiluminescence reagent (Thermo Scientific, Rockford, IL) was added and detected using Gel Doc™ XR ChemiDoc™ imaging system (BioRad, Hercules, CA) followed by quantification using ImageLab software (BioRad, Hercules, CA). Restore Plus Western Blot Buffer (Thermo Scientific, Rockford, IL) was used to strip membranes of antibodies prior to probing for other targets. Beta-Actin was used as a loading control to normalize densitometric values.

### siRNA knockdown

Knockdown of IGFBP-6 was done in DMEM supplemented with 10% fetal bovine serum, 1mM sodium pyruvate, and 2mM L-glutamine (Life Technologies, Carlsbad, CA). For immunoblot, 250,000 cells were plated per well in a 6-well plate. After 48 hours, the media was switched to charcoal-stripped DMEM containing the steroid treatment as described above, or the cells were harvested for analysis. Cells were then detached with 0.05 trypsin, suspended in maintenance media, and centrifuged at 1000 rpm for 5 min. The resulting pellet was then washed with 1X PBS and recentrifuged. After aspirating the PBS, the cell pellet was flash-frozen at-80 °C. Cell pellets were then stored at -80 °C until use. For FUCCI reporter assays, 12,500 T47D cells per well in a 96-well plate. The concentrations of siRNA and transfection reagent remained the same. Cells were then incubated for 48 hours before performing the corresponding assay. The sequences which target IGFBP-6 are listed as follows: siRNA #1 Sense: 5’-GGAGAAUCCUAAGGAGAGUtt-3’Anti-Sense: 5’-ACUCUCCUUAGGAUUCUCCtc-3’, #2 Sense: 5’-GAGGAGAAUCCUAAGGAGAtt-3’, Anti-Sense: 5’-UCUCCUUAGGAUUCUCCUCtg-3’, and #3 Sense: 5’-GAAUCCUAAGGAGAGUAAAtt-3’, Anti-Sense: 5’-UUUACUCUCCUUAGGAUUtc-3’. The sequences which target SH2D4A are as follows: siRNA A: Sense: 5’-CGAAGCAGAUUUGUAAGAGTT-3’, Anti-Sense: 5’-CUCUUACAAAUCUGCUUCGTT-3’, B: Sense: 5’-GGAGGAACCCAUCAUUCCTT-3’, Anti-Sense: 5’-GGAAGUGAUGGGUUCCUCCTT-3’, and C: Sense: 5’-CCAGGACAUGAGAGACAUATT-3’, Antisense: 5’-UAUGUCUCUCAUGUCCUGGTT-3’. Silencer™ Negative control #1 (Life Technologies, Carlsbad, CA) was used as a negative control at the same corresponding doses for each target.

### Data-independent acquisition proteomics

Total protein from each sample was reduced, alkylated, and purified by chloroform/methanol extraction prior to digestion with sequencing-grade modified porcine trypsin (Promega, Fitchburg, WI). Tryptic peptides were then separated by reverse phase XSelect CSH C18 2.5μm resin (Waters, Milford MA) on an in-line 150 x 0.075 mm column using an UltiMate 3000 RSLCnano system (Thermo Scientific, Rockford, IL). Peptides were eluted using a 60 min gradient from a 98:2 to a 65:35 buffer A:B ratio. Buffer A was composed of 0.1% formic acid, 0.5% acetonitrile, and buffer B was composed of 0.1% formic acid, 99.9% acetonitrile. Eluted peptides were ionized by electrospray (2.4 kV) through a heated capillary (275 °C) followed by data collection on an Orbitrap Exploris 480 mass spectrometer (Thermo Scientific, Rockford, IL).

Precursor spectra were acquired with a scan from 385–1015 Th at a resolution set to 60,000 with 100% AGC, max time of 50 msec, and an RF parameter at 40%. DIA was configured on the Orbitrap 480 to acquire 40 x 15 Th isolation windows, with normalized AGC target 500% and maximum injection time 40ms. A second DIA was acquired in a staggered window (15 Th) pattern with optimized window placements from 390–990 Th.

Following data acquisition, data were searched using Spectronaut (Biognosys version 19.1) against the UniProt Homo sapiens database (4th version of 2024) using the directDIA method with an identification precursor and protein q-value cutoff of 1%, generate decoys set to true, the protein inference workflow set to maxLFQ, inference algorithm set to IDPicker, quantity level set to MS2, cross-run normalization set to false, and the protein grouping quantification set to median peptide and precursor quantity. Protein MS2 intensity values were assessed for quality using ProteiNorm (22). The data were normalized using VSN (23) and analyzed using proteoDA, which performed statistical analysis using Linear Models for Microarray Data (limma) with empirical Bayes (eBayes) smoothing of the standard errors (24, 25). Proteins with an FDR-adjusted p-value less than 0.05 and a fold change greater than 1.5 were considered significant.

### Gene set enrichment analysis

The general method for gene set enrichment analysis is described by Subramanian et al. (26). Proteins were ranked by their relative fold change relative to the scrambled negative control. GSEA was performed using the fgsea R package (27). The Reactome pathway dataset was used to match proteins to pathways in the analysis (28).

## Results

### Progesterone alters multiple signaling pathways in breast cancer cells

**Figure 1:**
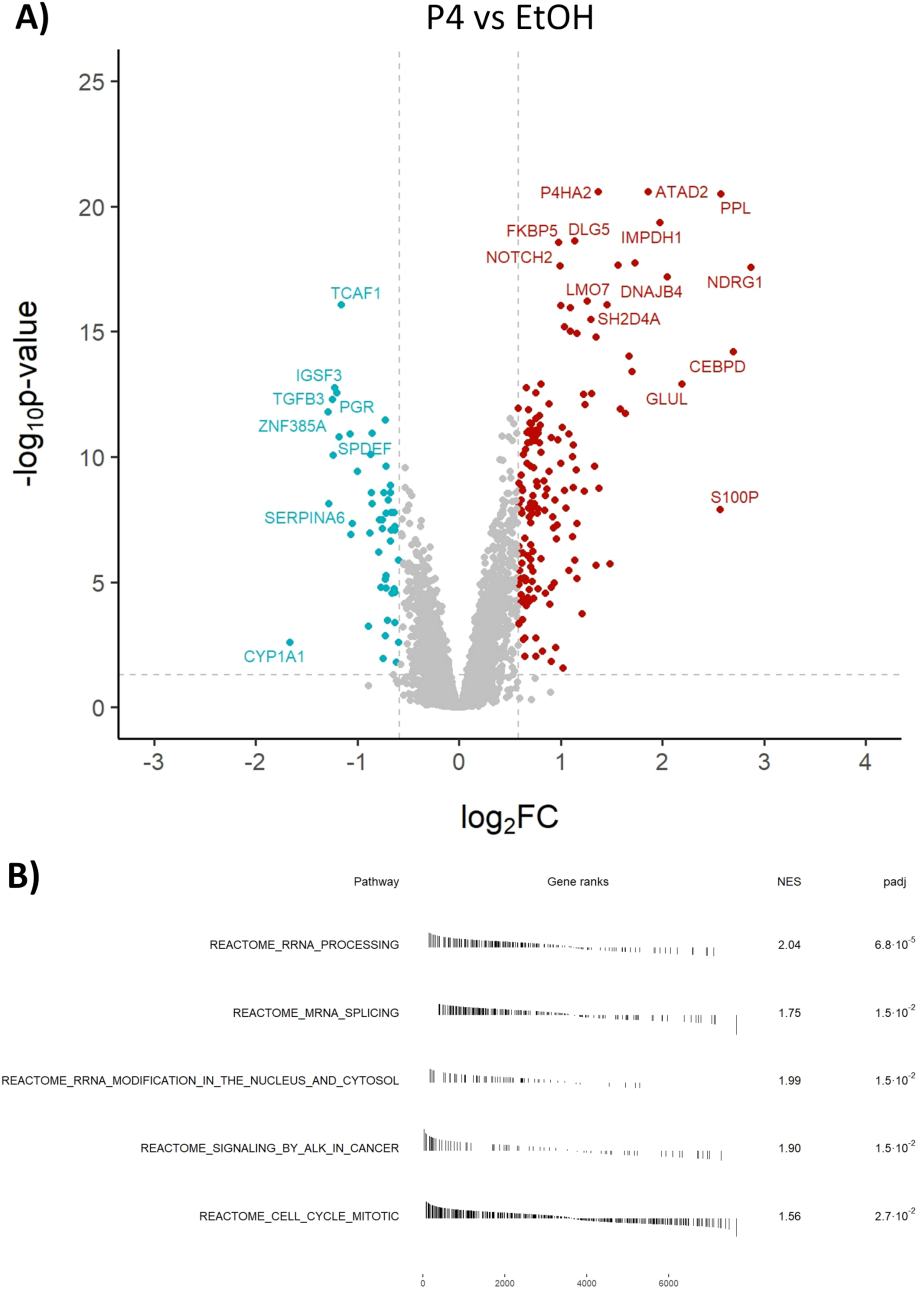
Differential protein expression following progesterone treatment. **(A)** Upregulated proteins are labeled in red and downregulated proteins are labeled in blue. All differentially expressed proteins are above a relative fold change of 1.5 and an adjusted p-value of 0.05. **(B)** Enriched pathways following progesterone treatment. Geneset enrichment analysis was conducted using reactome pathways.

IGFBP-6 was knocked down using siRNAs 1 and 3, as previously described for proteomics analysis, to block P4-induced IGFBP-6 induction (5). In total, 8638 unique peptides were identified. All data regarding quality control is presented in supplemental **Figures 1**–**4**. To determine the effects of P4, the negative control treated with progesterone was compared to cells treated with negative control and ethanol. 161 proteins were upregulated, and 54 proteins were downregulated (**Figure 1A**; **Supplementary Table 1**), which included Cytochrome P450 1A1 (CYP1A1), Corticosteroid-binding globulin (SERPINA6), Transforming growth factor beta 3 (TGFβ3), and SAM pointed domain-containing Ets transcription factor (SPDEF). The full list of differentially expressed proteins is listed in **Supplementary Table 1**. CYP1A1 is an enzyme associated with phase 1 metabolism of estrogen by adding a 2-hydroxyl group to E2 (29). Knockdown of CYP1A1 negatively impacts the proliferation of breast cancer cells independent of ERα status (30). This result suggests that progesterone also alters estrogen metabolism. SERPINA6 is mainly associated with cortisol availability in a manner that is regulated by progesterone (31). TGFβ3 has complex roles in tumorigenesis, and in breast cancer, has been associated with lymph node invasion (32). SPDEF has a controversial role in tumorigenesis but has been associated with poor prognosis in breast cancer (33).

**Figure 2:**
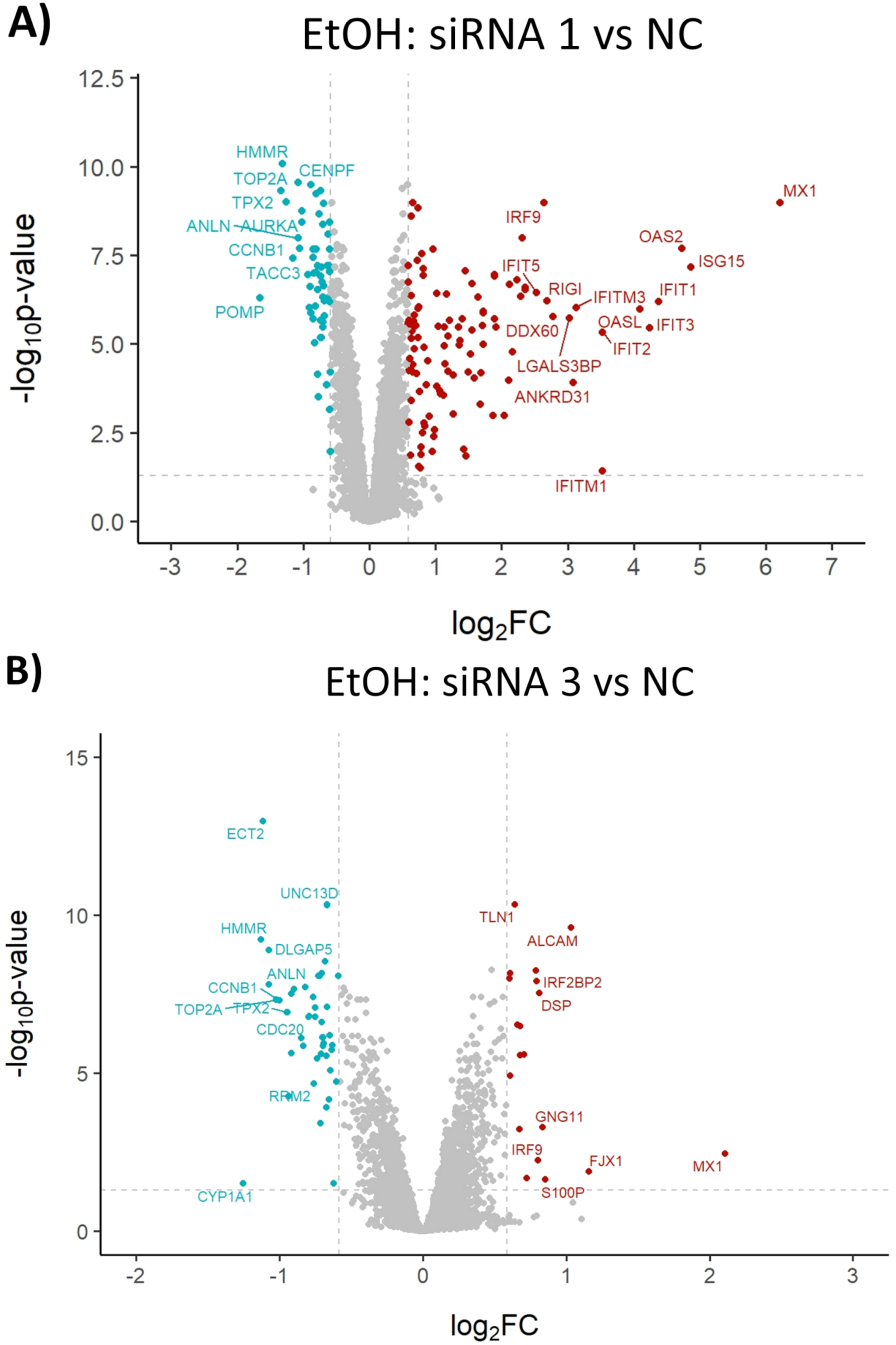
Differential protein expression following IGFBP-6 knockdown with siRNA 1. **(A)** Upregulated proteins are labeled in red and downregulated proteins are labeled in blue. **(B)** Differential Protein Expression Following IGFBP-6 Knockdown With siRNA 3. Upregulated proteins are labeled red and downregulated proteins are labeled in blue. All differentially expressed proteins are above a relative fold change of 1.5 and an adjusted p-value of 0.05.

**Figure 3:**
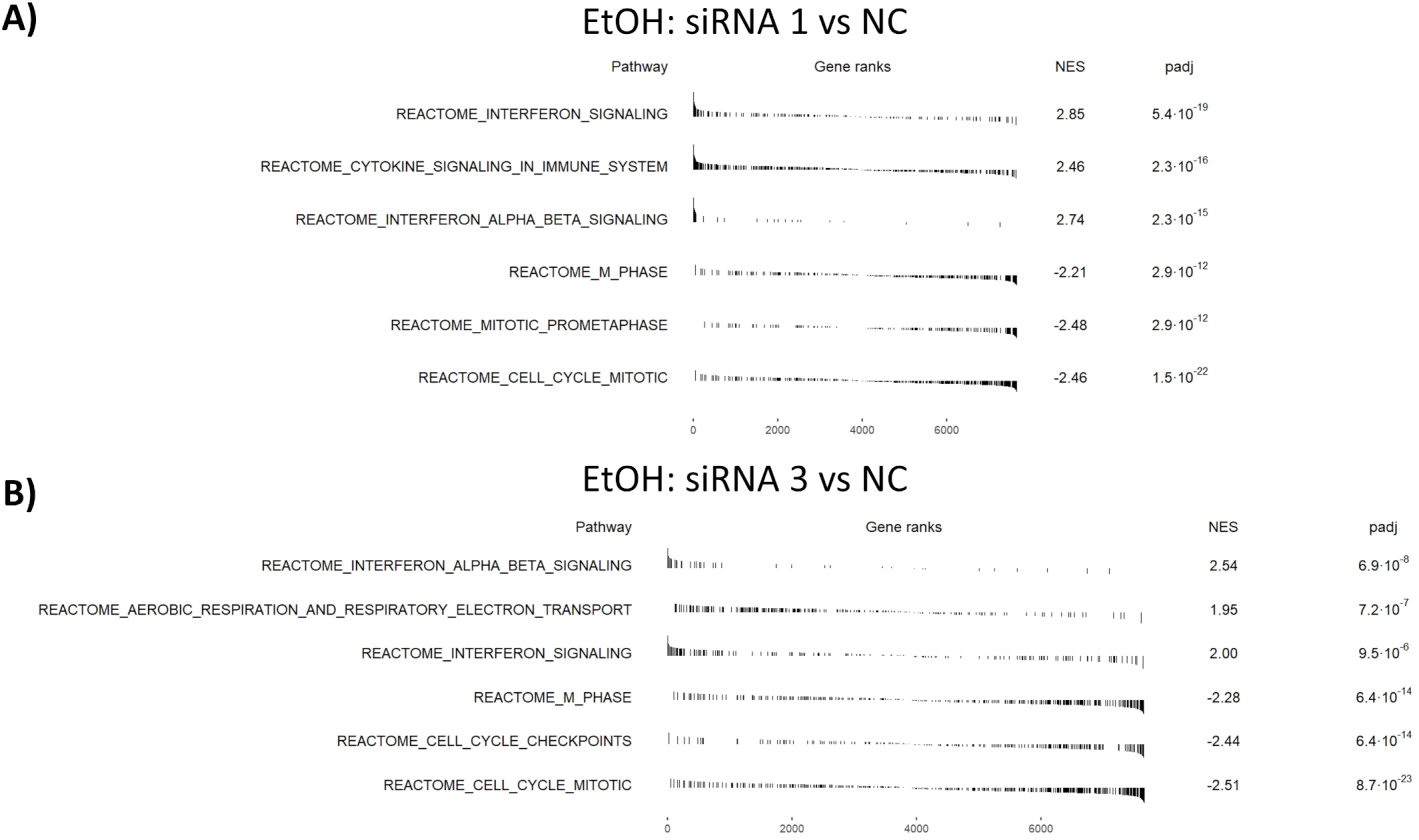
Enriched pathways following IGFBP-6 knockdown plus EtOH. **(A)** Enriched pathways following IGFBP-6 knockdown with ethanol with siRNA 1 relative to the negative control. **(B)** Following IGFBP-6 knockdown with ethanol with siRNA 3 relative to the negative control. Geneset enrichment analysis was conducted using reactome pathways.

**Figure 4:**
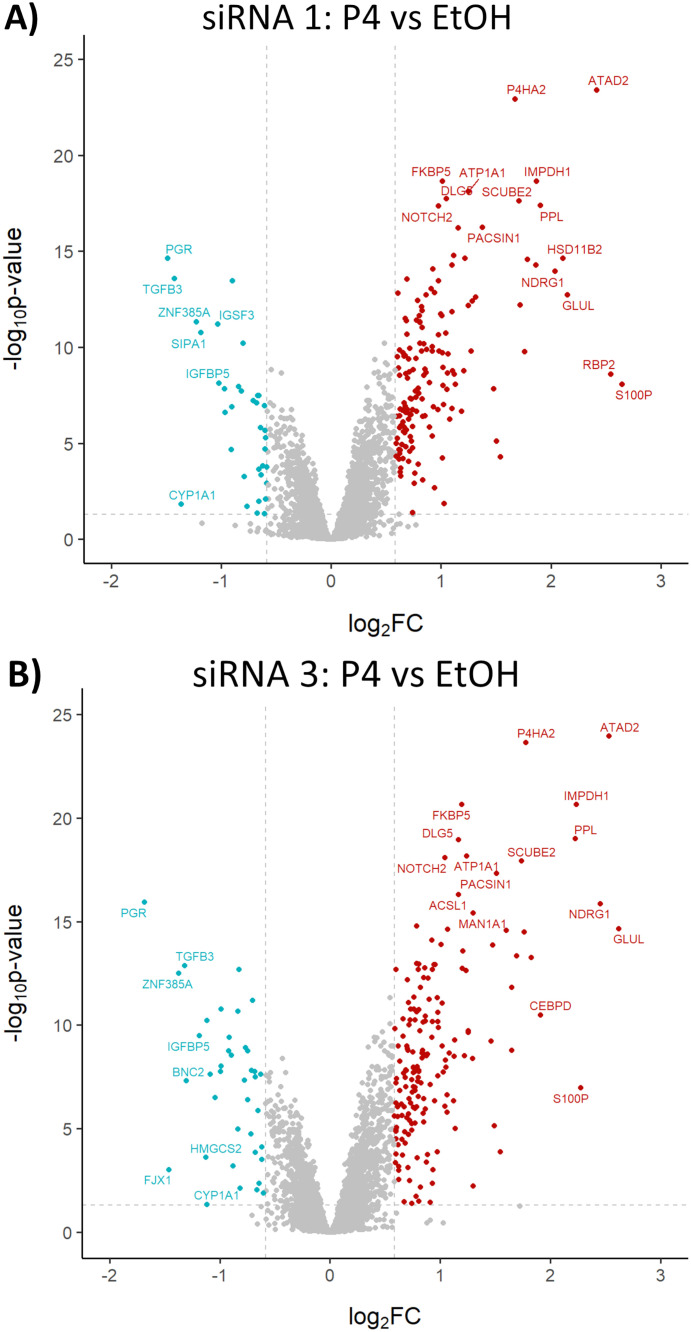
Differential protein expression comparing progesterone and ethanol after IGFBP-6 knockdown. **(A)** Differential protein expression comparing progesterone and ethanol after siRNA 1 treatment. Upregulated proteins are labeled red and downregulated proteins are labeled in blue. **(B)** Differential protein expression comparing progesterone and ethanol after siRNA 3 treatment. Upregulated proteins are labeled red and downregulated proteins are labeled blue. All differentially expressed proteins are above a relative fold change of 1.5 and an adjusted p-value of 0.05.

The most upregulated proteins included NDRG1, CCAAT/enhancer binding protein delta, (CEBPD), Periplakin (PPL), S100-P, and Glutamine synthetase (GLUL). NDGR1 is known to be upregulated by progesterone and to negatively regulate migration and the activation of AKT in breast cancer cells (34). CEBPD has been associated with ER/PR+ cancers and is a downstream effector of STAT3 (35). Periplakin is a protein associated with the cytoskeletal structure but has not been well studied (36). S100-P binds calcium and promotes proliferation, adhesion, migration, and invasion in breast cancer cells (37). GLUL is an enzyme that synthesizes glutamine from glutamate and promotes proliferation in HER2+ breast cancer cells (38).

To determine pathways impacted by progesterone, gene set enrichment analysis was performed using Reactome pathways. Only five significantly enriched pathways were identified (**Figure 1B**). Treatment of T47D cells with P4 is associated with increased rRNA processing, mRNA splicing, ALK signaling, and mitosis. Alk is overexpressed in aggressive breast cancer (39). Taken together, these results indicate that progesterone regulates proteins which participate in many diverse processes.

### IGFBP-6 regulates progression of the cell cycle

When IGFBP-6 was knocked down and treated with ethanol, 128 proteins were upregulated, and 61 were downregulated in siRNA 1. For siRNA 3, 33 proteins were upregulated, and 47 were downregulated. 35 proteins were significantly downregulated in both siRNA treatment, and only 9 proteins were upregulated. The differentially expressed proteins observed in both siRNAs are listed in **Supplementary Table 2**. Gene set enrichment analysis was performed to identify pathways associated with the differentially expressed proteins. In treatment with both siRNAs, Interferon signaling was upregulated, and pathways associated with the cell cycle were downregulated (**Figures 2**, **3**).

The most upregulated proteins in both siRNA treatments included MX1, Interferon Regulatory Factor 9 (IRF9), S100P, 14-3–3 eta, and ATP1B1 (**Figures 2A, B**). The most downregulated proteins included Topoisomerase 2 alpha (TOP2A), Hyaluronan-mediated motility receptor (HMMR), TPX2, TACC3, and Centromere Protein F. These proteins are associated with the G2/M phase of the cell cycle (40). HMMR and IRF9 are regulated by IGFBP-6 knockdown and have been described previously (19). HMMR associates with TPX2, which in turn associates with Aurora kinase to regulate microtubule formation (41). IRF9 was discussed as part of the ISGF3 complex, which promotes interferon signaling (42). 14-3–3 eta (YWHAH) promotes metastasis in breast cancer (43). ATP1B1 is an ATP-dependent Na+/K+ transporter that negatively regulates the invasion and migration of breast cancer cells (44).

**Figure 5:**
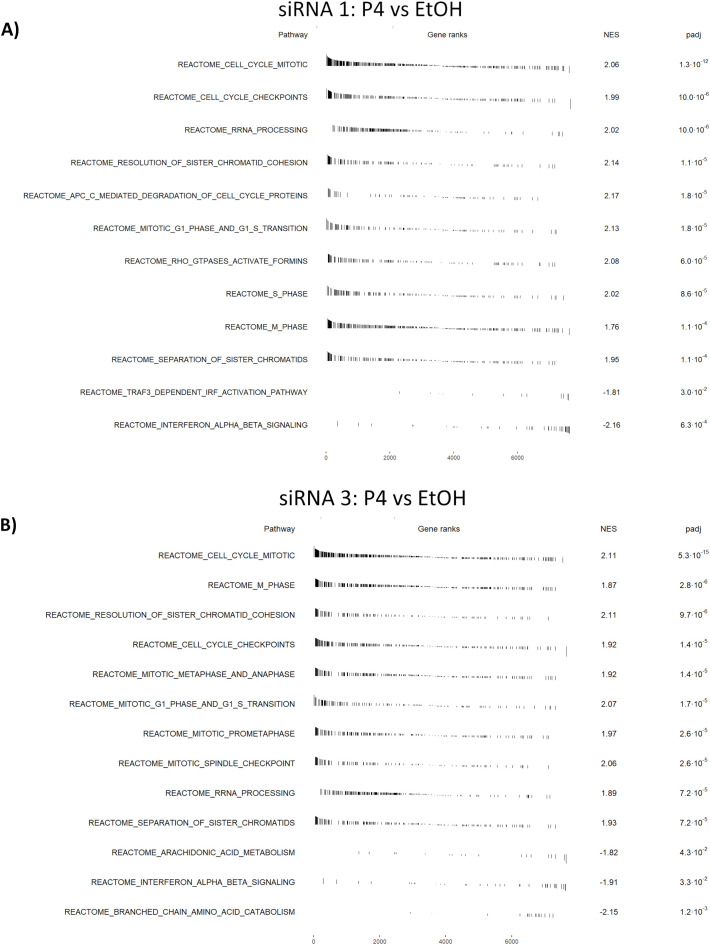
Enriched pathways comparing progesterone and ethanol after IGFBP-6 knockdown. **(A)** Enriched pathways comparing progesterone and ethanol after siRNA 1 treatment. **(B)** Enriched pathways comparing progesterone and ethanol after siRNA 3 treatment. Gene set enrichment analysis was conducted using reactome pathways.

For each siRNA, differential protein expression was compared in cells treated with P4 versus the vehicle (**Figures 4A, B**). Differential protein expressions were very similar to those presented in the previous sections describing the comparison between progesterone and the vehicle (**Figure 1**; **Supplementary Table 3**). Another member of the IGFBP family, IGFBP-5, was negatively regulated by P4. Gene set enrichment analysis indicates that in IGFBP-6 low cells, treatment with progesterone increases the levels of proteins associated with events that occur in mitosis, such as the mitotic spindle checkpoint and separation of sister chromatids (**Figures 5A, B**). These results further suggest that IGFBP-6 promotes progression into mitosis in response to progesterone.

### IGFBP-6 alters expression of proteins induced by progesterone

**Figure 6:**
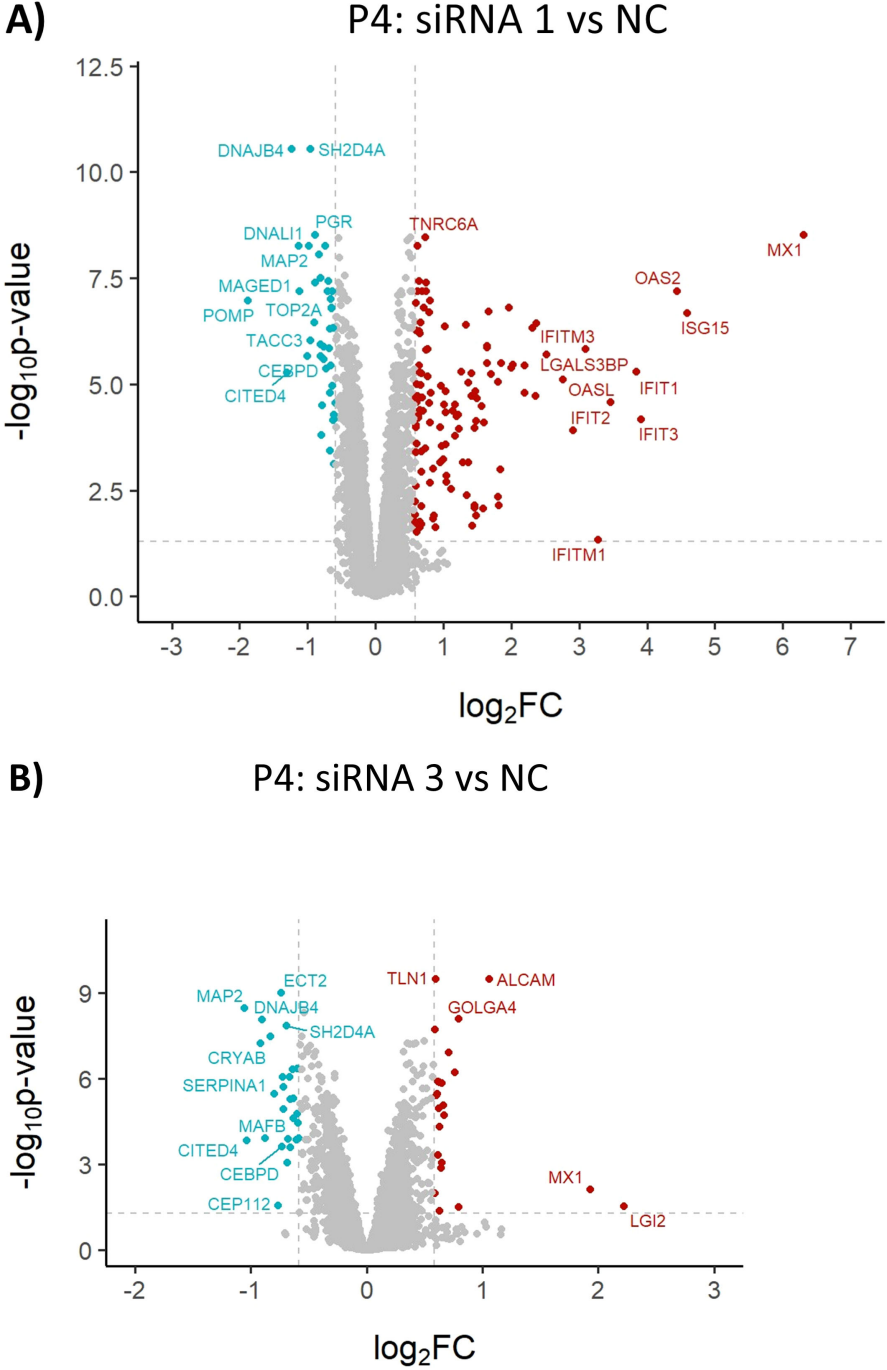
Differential protein expression comparing IGFBP-6 knockdown Vs the negative control in progesterone-treated cells. **(A)** Differential protein expression comparing siRNA 1 and the negative control with progesterone treatment. **(B)** Differential protein expression comparing siRNA 3 and the negative control with progesterone treatment. Upregulated proteins are labeled red, and downregulated proteins are labeled in blue. All differentially expressed proteins are above a relative fold change of 1.5 and an adjusted p-value of 0.05.

In a previous study (5), it was demonstrated that IGFBP-6 modulates the levels of progesterone receptor and is required for some of the downstream actions of progesterone in T47D cells. Following treatment with progesterone, comparisons between IGFBP-6 knockdowns and the scrambled negative control are shown in **Figures 6A, B**. Upregulated proteins included MX1, ATP1B1, 14-3–3 Eta, and ALCAM. Downregulated proteins include but are not limited to Cbp/p300-interacting transactivator 4 (CITED4), DNAJB4, DNALI1, CEBPD, and SH2D4A. In total, 17 proteins were downregulated, and 9 were upregulated following treatment with IGFBP-6 targeting siRNAs with progesterone treatment. **Table 1** lists all the differentially expressed proteins after treatment with P4.

**Table 1:** IGFBP-6 knockdown vs negative control in progesterone treated cells.

Upregulated			siRNA_A_P4_vs_NC_P4		siRNA_B_P4_vs_NC_P4	
Protein	Uniprot id	Description	logFC	adj.P.Val	logFC_	adj.P.Val
MX1	P20591	Interferon-induced GTP-binding protein Mx1	6.306192	3.027E-09	1.92857	0.00759025
ATP1B1	P05026	Sodium/potassium-transporting ATPase subunit beta-1	0.789157	2.048E-07	0.76343	6.1185E-07
QKI	Q96PU8	KH domain-containing RNA-binding protein QKI	0.768496	1.463E-06	0.66653	1.9769E-05
YWHAH	Q04917	14-3–3 protein eta	0.751006	4.099E-08	0.71168	1.2088E-07
ALCAM	Q13740	CD166 antigen	0.741931	6.338E-08	1.05992	3.2795E-10
ZDHHC20	Q5W0Z9	Palmitoyltransferase ZDHHC20	0.716704	1.606E-07	0.60561	4.0043E-06
SPDEF	O95238	SAM pointed domain-containing Ets transcription factor	0.671269	1.03E-05	0.62926	4.8344E-05
SCAMP1	O15126	Secretory carrier-associated membrane protein 1	0.61815	5.448E-09	0.58882	1.9066E-08
CLIC4	Q9Y696	Chloride intracellular channel protein 4	0.59098	2.059E-05	0.66292	8.8296E-06
Downregulated						
Protein	Uniprot id	Description	logFC	adj.P.Val	logFC	adj.P.Val
CITED4	Q96RK1	Cbp/p300-interacting transactivator 4	-1.306002	5.375E-06	-1.0361	0.00014837
DNAJB4	Q9UDY4	DnaJ homolog subfamily B member 4	-1.233982	2.866E-11	-0.9022	8.9884E-09
DNALI1	O14645	Axonemal dynein light intermediate polypeptide 1	-1.126449	5.448E-09	-0.5864	0.00012466
MAGED1	Q9Y5V3	Melanoma-associated antigen D1	-1.122221	6.338E-08	-0.6763	0.00012979
CEBPD	P49716	CCAAT/enhancer-binding protein delta	-1.008084	2.188E-06	-0.7338	0.00024222
MAP2	P11137	Microtubule-associated protein 2	-0.985998	5.448E-09	-1.0534	3.5453E-09
TACC3	Q9Y6A5	Transforming acidic coiled-coil-containing protein 3	-0.954888	9.479E-07	-0.658	0.00025914
SH2D4A	Q9H788	SH2 domain-containing protein 4A	-0.953534	2.866E-11	-0.6924	1.4443E-08
TOP2A	P11388	DNA topoisomerase 2-alpha	-0.895795	3.576E-07	-0.6079	0.00014063
PGR	P06401	Progesterone receptor	-0.892596	3.027E-09	-0.6416	4.8905E-07
HMMR	O75330	Hyaluronan mediated motility receptor	-0.885013	4.057E-08	-0.6574	5.3781E-06
MAFB	Q9Y5Q3	Transcription factor MafB	-0.792834	0.0001587	-0.8773	0.00012462
CDC20	Q12834	Cell division cycle protein 20 homolog	-0.765692	2.603E-06	-0.7192	1.163E-05
PALMD	Q9NP74	Palmdelphin	-0.692884	3.797E-08	-0.6021	4.4665E-07
DLGAP5	Q15398	Disks large-associated protein 5	-0.686011	1.401E-06	-0.6007	1.6609E-05
CRYAB	P02511	Alpha-crystallin B chain	-0.662376	3.626E-06	-0.9135	5.9929E-08
SERPINA1	P01009	Alpha-1-antitrypsin	-0.616566	5.189E-05	-0.7955	3.4922E-06

MX1 is an interferon-stimulated gene (ISG) whose expression increases following IGFBP-6 knockdown (19). ATP1B1 is induced by progesterone, but at higher levels when IGFBP-6 is low. ALCAM is associated with cell adhesion and has been identified as a potential biomarker predicting worse outcomes for breast cancer patients (45). Cited4 associates with p300 to prevent its association with HIF1a (46) and binds AP-2 to weakly promote its association with p300, thereby altering gene expression (47). DNAJB4 is a member of the 40 kDa Heat shock protein family and is an emerging biomarker for breast cancer with roles as a tumor suppressor (48–50). DNALI1 has an unknown role in breast cancer but has been associated with poor prognoses in low-grade glioma (51). CEBPD was induced by progesterone, but at lower levels when IGFBP-6 is low.

**Figure 7:**
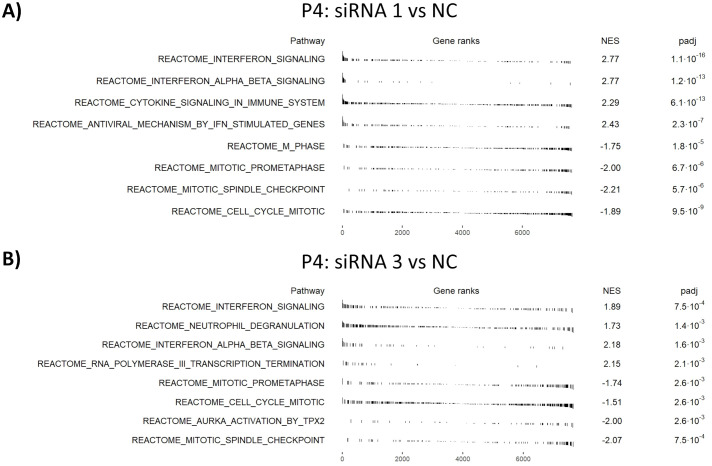
Enriched pathways In IGFBP-6 low cells treated with progesterone. **(A)** Enriched pathways comparing siRNA 1 and the negative control after progesterone treatment. **(B)** Enriched pathways comparing siRNA 3 and the negative control after progesterone treatment. Gene set enrichment analysis was conducted using reactome pathways.

Gene set enrichment analysis demonstrates that Interferon signaling is active in IGFBP-6 low cells and pathways associated with the G2/M phase of the cell cycle are downregulated (**Figures 7A, B**). These results indicate that IGFBP-6 is induced by P4 to promote entry into M phase.

### Progesterone induces SH2D4A by upregulation of IGFBP-6

**Figure 8:**
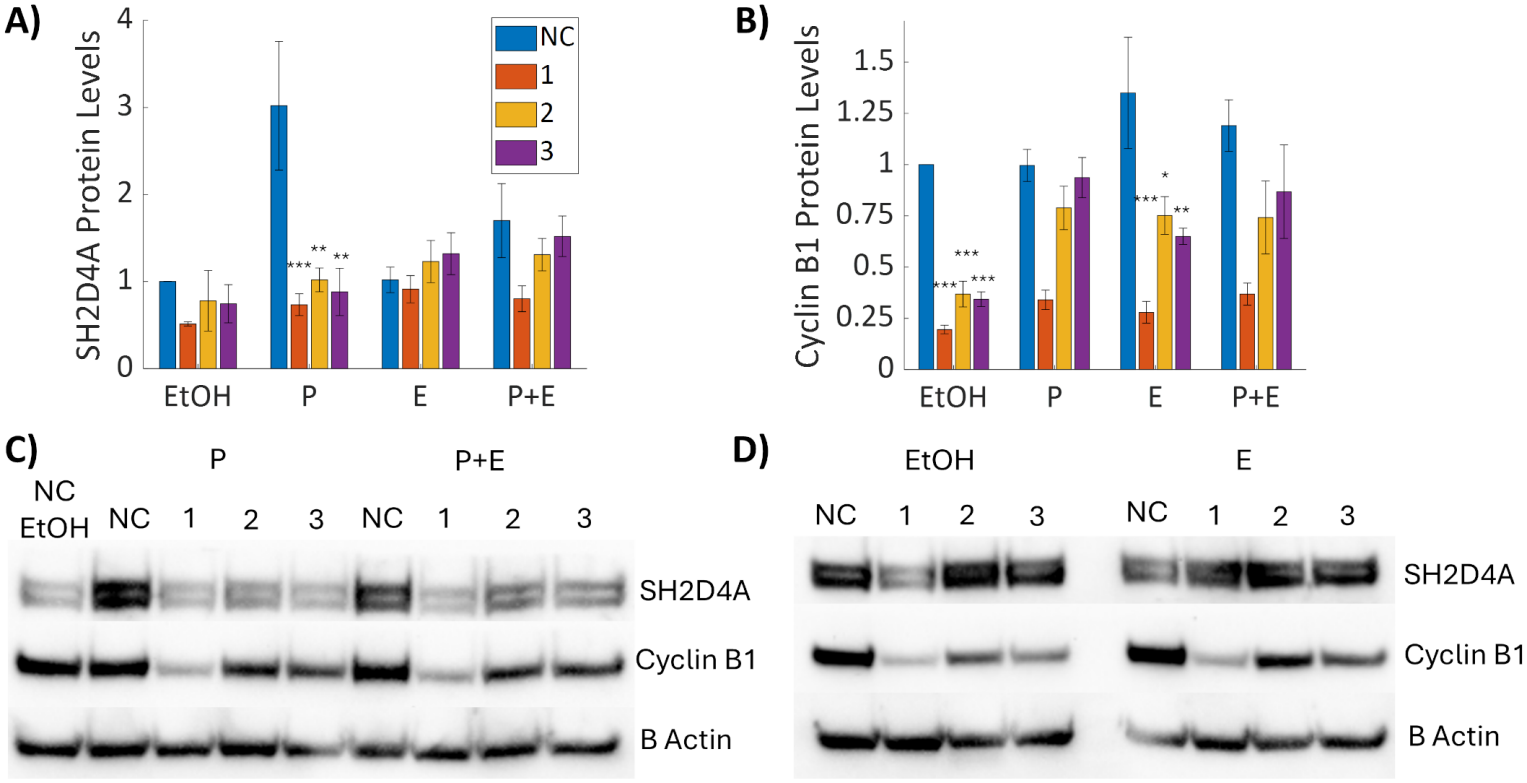
SH2D4A and Cyclin B1 Levels Following IGFBP-6 Knockdown. **(A)** SH2D4A levels relative to the negative control plus ethanol. Results of a two-way ANOVA on log-transformed data are as follows: siRNA Treatment p<0.001, hormone treatment p<0.05, and for the interaction p <0.05. **(B)** Cyclin B1 levels relative to the negative control plus ethanol. Results of the two-way ANOVA are as follows: siRNA Treatment p<0.001, hormone treatment p<0.001, and for the interaction p <0.05. **(C)** Representative western blot for SH2D4A and Cyclin B1 levels after treatment with progesterone (P) or progesterone plus estradiol (P+E). **(D)** Representative western blot for SH2D4A and Cyclin B1 levels after treatment with ethanol (EtOH) or estradiol **(E)**. All asterisks represent statistical significance relative to ethanol. *means p< 0.05, **means p<0.01, and ***means p<0.001.

SH2D4A was identified in the proteomics data as a protein whose levels were increased by progesterone (**Figure 1A**) but would decrease in IGFBP-6 low cells (**Figures 6A, B**). The results reported in the proteomics data were validated by western blot (**Figure 8**). Previously, it was reported that IGFBP-6 was necessary for progesterone to antagonize estradiol’s actions, but no mechanism was identified (5). SH2D4A has been shown to bind Estrogen Receptor and inhibit proliferation (52). This result suggests that IGFBP-6 antagonizes ER activation by regulating SH2D4A expression. The effects of low IGFBP-6 on SH2D4A were most apparent when cells were treated with progesterone (**Figures 8A, C**). When IGFBP-6 is induced by progesterone, SH2D4A levels increase 3-fold. Knockdown of IGFBP-6 did not significantly affect SH2D4A levels when cells were treated with ethanol, estradiol, or both progesterone and estradiol. It is not clear why cotreatment with progesterone and estradiol reduced SH2D4A induction. SH2D4A has other roles, including inhibition of interleukin-6 signaling by preventing STAT3 nuclear localization (53), centrosome maturation, and chromatid segregation (54).

Cyclin B1 was also measured (**Figures 8B, D**) and was consistent with prior studies (19). The knockdown of IGFBP-6 resulted in a significant decrease in cyclin B1 when cells were treated with ethanol or estradiol. When treated with progesterone or progesterone and estradiol, no consistent significant results were observed with IGFBP-6 knockdown, where levels of cyclin B1 were restored. These results suggest that IGFBP-6 promotes the accumulation of cyclin B1 in the absence of progesterone.

**Figure 9:**
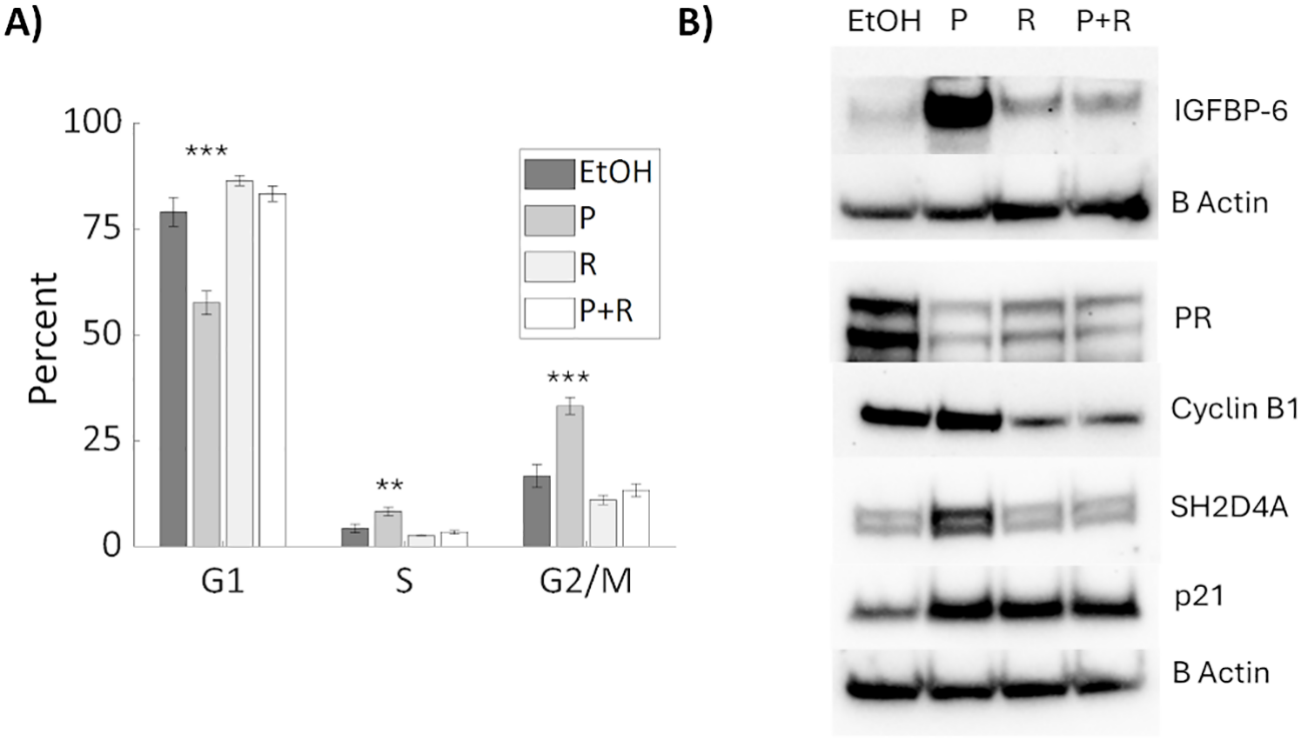
Effects of progesterone receptor inhibition on the cell cycle. **(A)** Relative cell cycle phase distribution after progesterone receptor inhibition. **(B)** Representative western blots for IGFBP-6, PR, Cyclin B1, SH2D4A, and P21 for cells treated with ethanol (EtOH), Progesterone (P), RU-486 (R), or both (P+R). ANOVA results are as follows: for G1 p<0.001, for S p<0.01, and for G2/M p<0.001. All asterisks represent statistical significance relative to ethanol. **means p<0.01, and ***means p<0.001.

To further examine the effects of progesterone signaling on SH2D4A and cyclin B1, mifepristone (RU-486), a PR inhibitor, was used to measure effects on the cell cycle. T47D cells transfected with a FUCCI reporter were used to determine effects on cell cycle distributions (**Figure 9A**). Treatment with progesterone results in a lower percentage of cells in G1 and an increase in the percentage of cells in G2/M. Inhibition of PR with RU-486 alone or in combination with progesterone arrests cells in G1. These results agree with previously reported studies (19). Inhibition of PR also results in decreased levels of PR and Cyclin B1, along with no induction of IGFBP-6 or SH2D4A (**Figure 9B**). P21, another progesterone-responsive protein, is induced by both progesterone and RU-486. All densitometry associated with these blots can be found in Supplemental **Figure 5**. These results demonstrate that progesterone promotes expression of cell cycle regulators as well as regulation of IGFBP-6 and SH2D4A.

**Figure 10:**
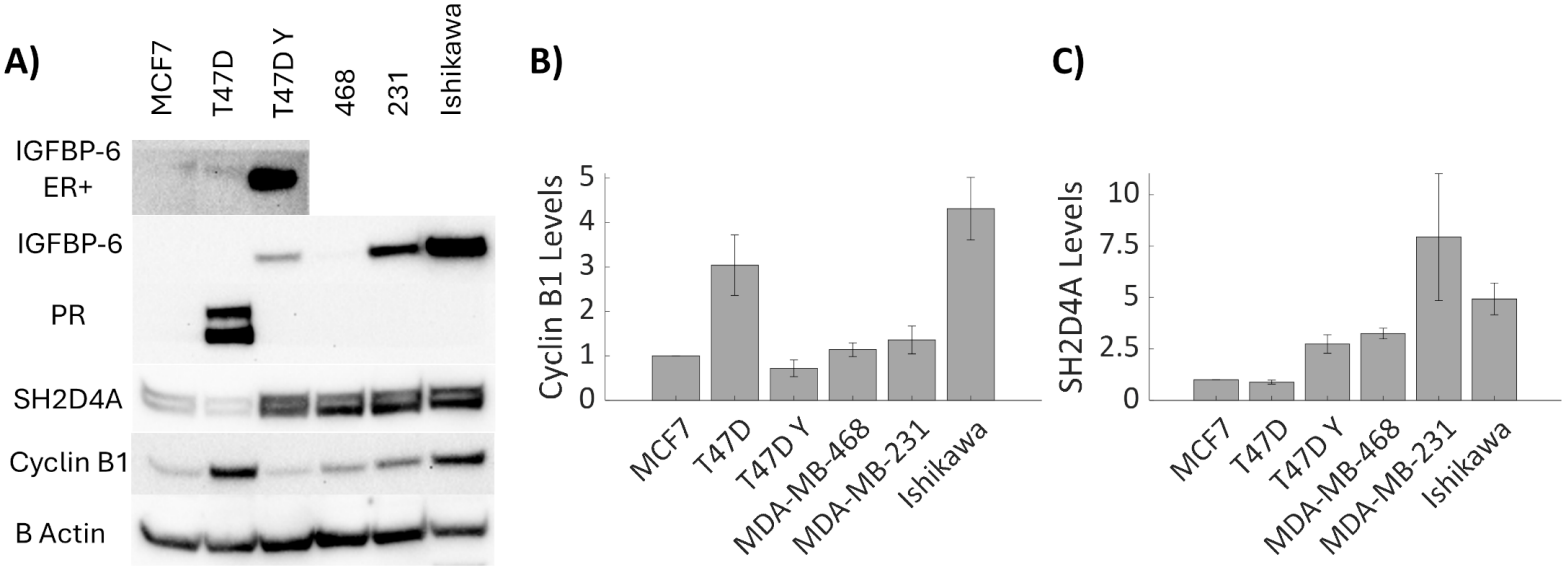
Cell line comparisons across five breast cancer cell lines and one endometrial cancer cell line. **(A)** Relative IGFBP-6, PR, SH2D4A, and Cyclin B1. **(B)** Relative Cyclin B1 levels normalized to expression in MCF-7 cells. **(C)** Relative SH2D4A levels normalized to expression in MCF-7 cells.

To further investigate the correlation between IGFBP-6 and SH2D4A, levels of both proteins were measured in other breast cancer cell lines and the Ishikawa endometrial cancer cell line (**Figure 10**). The lowest expression of IGFBP-6 in breast cancer cells is observed in MCF-7 cells, and the highest expression is observed in MDA-MB-231 cells. Ishikawa cells, an endometrial cancer cell line, expressed the highest levels of intracellular IGFBP-6. Cyclin B1 is strongly expressed in T47D compared to other cell lines and appears to be associated with PR levels (**Figures 10A, B**). SH2D4A levels positively correlate with IGFBP-6 in all cell lines (10C). Along with decreased SH2D4A levels in IGFBP-6-low cells, these results suggest that IGFBP-6 regulates SH2D4A levels and that progesterone-induced SH2D4A induction is IGFBP-6-mediated.

### Knockdown of SH2D4A results in cell cycle arrest at G1 and decreases in p21 accumulation

**Figure 11:**
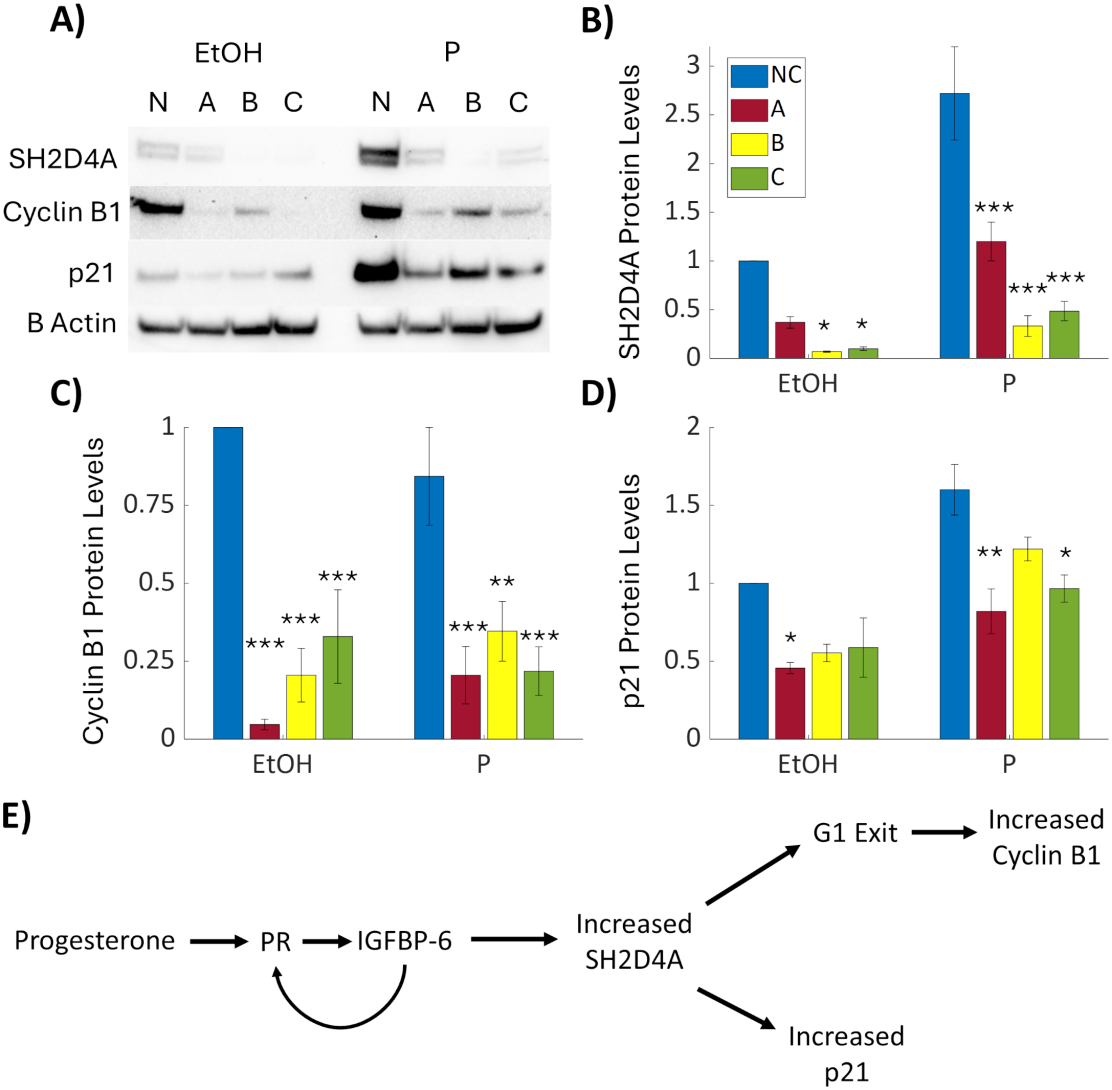
Knockdown of SH2D4A. **(A)** Representative western blot of SH2D4A, Cyclin B1 and p21 after SH2D4A knockdown with ethanol (EtOH) or Progesterone (P). **(B)** Relative SH2D4A levels after using three unique siRNA sequences that target SH2D4A. The results of the two-way ANOVA are as follows: for the siRNA treatment p<0.001, the hormone treatment p<0.001, and for the interaction term p<0.01. **(C)** Relative Cyclin B1 levels after SH2D4A knockdown with ethanol (EtOH) or Progesterone (P). The results of the two-way ANOVA are as follows: for the siRNA treatment p<0.001, the hormone treatment was not significant, and the interaction term was not significant. **(D)** Relative p21 levels after SH2D4A knockdown with ethanol (EtOH) or Progesterone (P). The results of the two way ANOVA are as follows: for the siRNA treatment p<0.001, the hormone treatment p<0.001, and the interaction term was not significant. All asterisks represent statistical significance relative to the negative control plus ethanol. *means p< 0.05, **means p<0.01, and ***means p<0.001. **(E)** Proposed Mechanism. Progesterone induces IGFBP-6 which in turn promotes SH2D4A expression. This promotes expression of p21 and G1 Exit.

To further investigate the role of SH2D4A in breast cancer cells, SH2D4A was knocked down using three unique sequences. The knockdown of SH2D4A was designed to prevent progesterone-induced induction, and siRNA B and C were the most efficient (**Figures 11A, B**). Since SH2D4A has been associated with regulating events in G2/M (54), Cyclin B1 was measured to determine if cell cycle progression was affected by lowered levels of SH2D4A. Accumulation of cyclin B1 decreases in SH2D4A low cells regardless of steroid hormone treatment (**Figure 11C**). P21 accumulation was also measured since it is also a progesterone-responsive gene (14, 16, 55). Previously, the IGFBP-6 knockdown did not induce p21 by progesterone (5). After knocking down SH2D4A, p21 levels decreased regardless of steroid hormone treatment (**Figure 11D**). These and previous results suggest that IGFBP-6 is part of a pathway that regulates SH2D4A accumulation and promotes progesterone-induced p21 induction.

**Figure 12:**
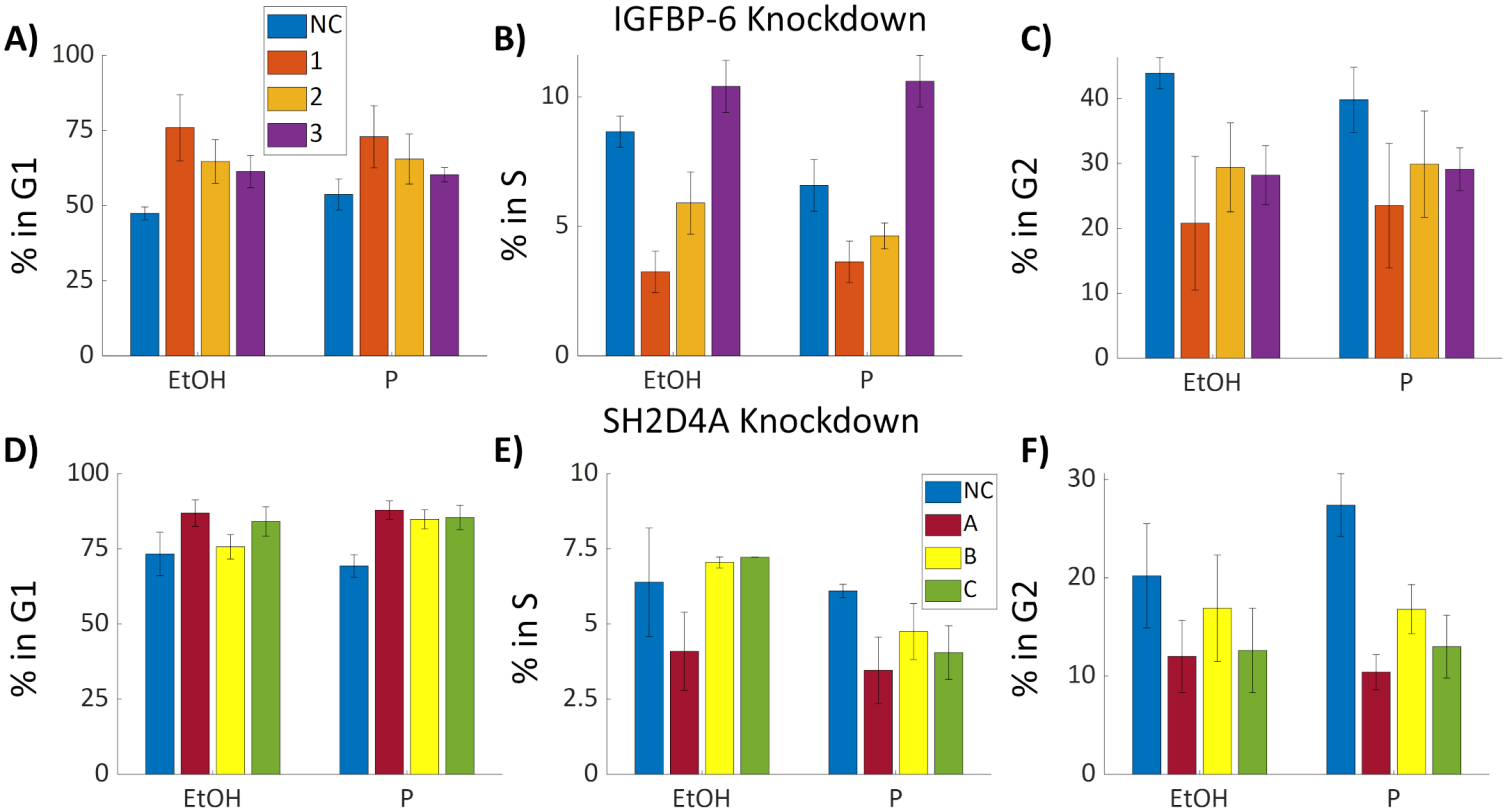
Effects on the cell cycle after IGFBP-6 or SH2D4A knockdown. **(A)** Percent of T47D-Fucci cells in G1 phase after IGFBP-6 knockdown ethanol (EtOH) or Progesterone (P). The results of the two-way ANOVA are as follows: for the siRNA treatment p<0.001, the hormone treatment p<0.05, and the interaction term was not significant. **(B)** Percent of T47D-Fucci cells in S phase after IGFBP-6 knockdown. The results of the two-way ANOVA are as follows: for the siRNA treatment p<0.001, the hormone treatment p<0.001, and the interaction had p<0.001. **(C)** Percent of T47D-Fucci cells in G2/M phases after IGFBP-6 knockdown. The results of the two-way ANOVA are as follows: for the siRNA treatment, p<0.001; the hormone treatment was nonsignificant, and the interaction term was not significant. **(D)** Percent of T47D-Fucci cells in G1 phase after SH2D4A knockdown. The results of the two-way ANOVA are as follows: for the siRNA treatment p<0.001, the hormone treatment was not significant, and the interaction term was not significant. **(E)** Percent of T47D-Fucci cells in S phase after SH2D4A knockdown. The results of the two-way ANOVA are as follows: for the siRNA treatment p<0.001, the hormone treatment was not significant, and the interaction term was not significant. **(F)** Percent of T47D-Fucci cells in G2/M phase after SH2D4A knockdown. The results of the two-way ANOVA are as follows: for the siRNA treatment p<0.001, the hormone treatment was not significant, and the interaction term was not significant.

Prior work has demonstrated that decreased levels of IGFBP-6 decrease breast cancer cell entry into G2/M (18). When IGFBP-6 low cells were treated with progesterone, a similar result was obtained to the knockdown of SH2D4A (**Figure 12**). IGFBP-6 low cells accumulated in G1 and decreased entry into G2/M regardless of hormone treatment, especially without progesterone (18) (**Figures 12A–C**). T47D-FUCCI cells had SH2D4A knocked down and were then treated with progesterone to measure effects on the cell cycle (**Figures 12D–F**). The knockdown of SH2D4A increases the percentage of cells in G1 and decreases the percentage of cells in G2 independently of steroid hormone treatment. These results suggest that IGFBP-6 and SH2D4A regulate cell cycle progression in similar ways and are part of the same signaling pathway.

Previously, it was determined that the knockdown of IGFBP-6 decreased PR and p21 levels while increasing cyclin E2 (5). Taken together with the results presented here, IGFBP-6 appears to regulate the levels or activity of PR, which, in turn, promotes exit from G1. In addition to IGFBP-6 knockdown, inhibition of PR and SH2D4A knockdown also arrest cells in G1. These results suggest the following mechanism. Progesterone activates PR, which induces IGFBP-6 and progression into the cell cycle. IGFBP-6 promotes SH2D4A accumulation, which in turn promotes exit of G1 and expression of Cyclin B1 as well as expression of p21 (**Figure 11E**).

### SH2D4A is protective in luminal a breast cancer

**Figure 13:**
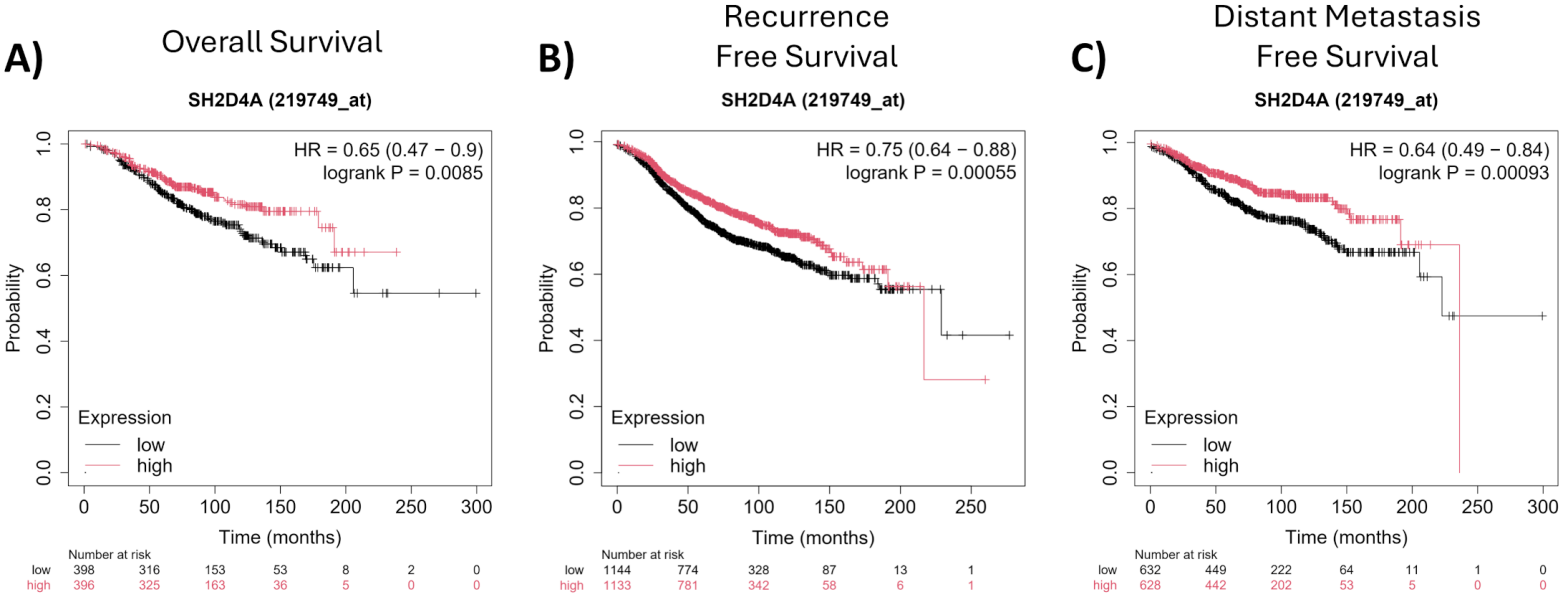
Kaplan meier curves for SH2D4A in luminal a breast cancer **(A)** overall survival. **(B)** Recurrence free survival **(C)** Distant metastasis free survival. All curves were generated using the Kaplan-Meier Plotter Tool with data from patients with luminal A breast cancer and divided by median expression of SH2D4A. The curve in red is for patients with expression over the median. The black curve is for patients with SH2D4A expression below the median.

To determine whether SH2D4A had any role in patient outcomes in breast cancer, Kaplan Meier Plots were generated using the KMPlotter tool (kmplot.com) using patient data obtained from the gene expression omnibus (GEO) and the European genome archive (EGA). When selecting patients with Luminal A breast cancer, SH2D4A improves overall survival, recurrence free survival, and distant metastasis free survival (**Figures 13A–C**). Overall survival had a hazard ratio of 0.65 (logrank P = 0.0085), recurrence free survival had a hazard ratio of 0.75 (logrank P = 0.00055) and distant metastasis free survival had a hazard ratio of 0.64 (logrank P = 0.00093). In previous studies, IGFBP-6 has been shown to confer a similar protective effect in ER positive and PR positive breast cancers (4–6). Taken together, these results suggest that IGFBP-6 may confer protection to patients in part by regulating SH2D4A levels.

### IGFBP-6, ER, and PR are regulated in a cell cycle phase dependent manner

**Figure 14:**
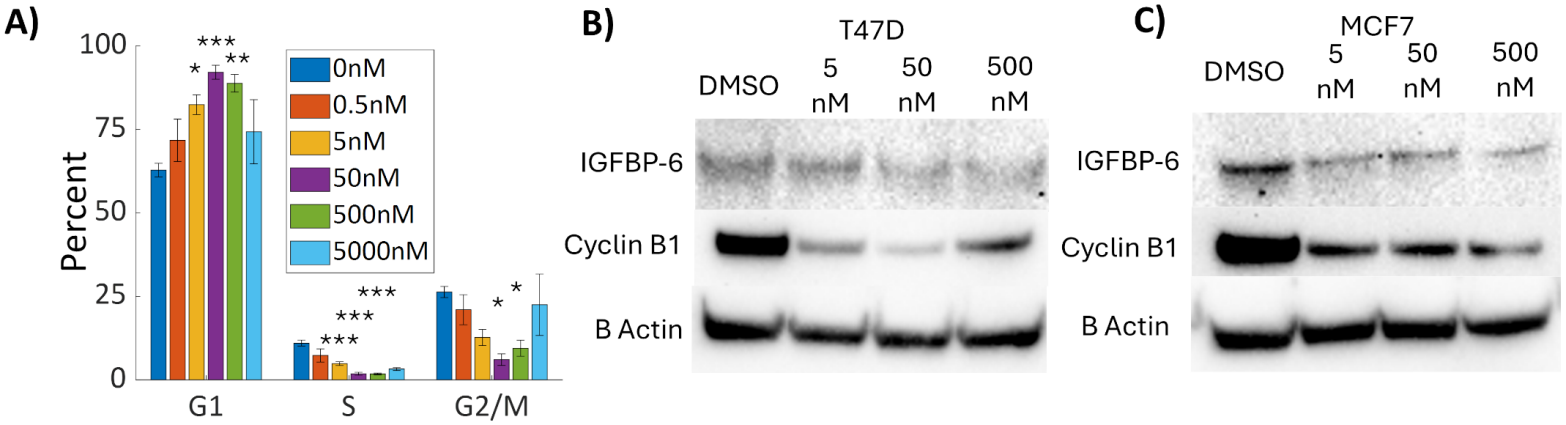
Arresting cells in G1 with abemaciclib decreases intracellular IGFBP-6 expression. **(A)** Cell cycle phase distributions of T47D-Fucci cells. The ANOVA results are as follows: for G1 p<0.01, for S p<0.001, and for G2/M p<0.01. All asterisks represent statistical significance relative to cells treated with DMSO. *means p< 0.05, ***means p<0.01, and ***means p<0.001. **(B)** Intracellular IGFBP-6 in T47D cells after treatment with varying doses of Abemaciclib. **(C)** Intracellular IGFBP-6 in MCF-7 cells after treatment with varying doses of Abemaciclib.

**Figure 15:**
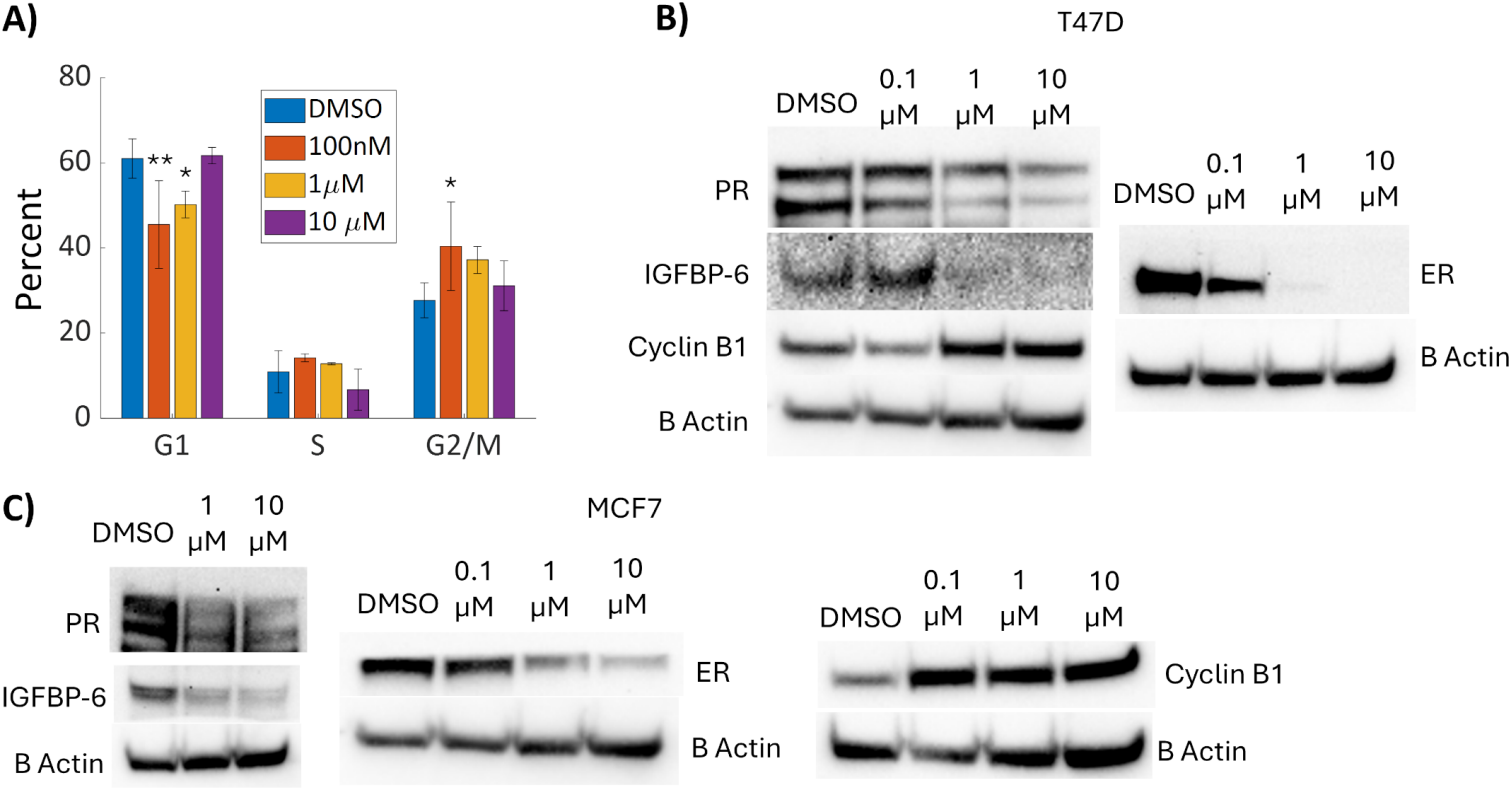
Arresting Cells in G2/M with Nocodazole Decreases ER, PR, and Intracellular IGFBP-6. **(A)** ER, PR, and intracellular IGFBP-6 decrease in T47D cells following high doses of nocodazole. **(B)** ER, PR, and intracellular IGFBP-6 decrease in MCF-7 cells following high doses of nocodazole. **(C)** Cell cycle phase distributions following nocodazole treatment. The ANOVA results are as follows: for G1 p<0.001, for S p<0.05, and for G2/M p<0.05. All asterisks represent statistical significance relative to cells treated with DMSO. *means p< 0.05 and **means p<0.01.

The results presented in previous sections demonstrate that IGFBP-6 is associated with the regulation of the cell cycle. To further investigate the activity of IGFBP-6 during cell cycle progression, cells were arrested during G1 with abemaciclib and G2/M using nocodazole (**Figures 14**, **15** and **Supplementary Figures 6**–**8**). After treating both T47D and MCF-7 cells with abemaciclib, which led to cell cycle arrest in G1 (**Figure 14A**), intracellular IGFBP-6 significantly decreased at 50nM and 500nM doses (**Figures 14B, C**). SH2D4A was not significantly changed under these conditions in MCF7 or T47D cells (**Supplementary Figure 9**). Extracellular IGFBP-6 was not significantly affected in all cell lines and was not affected intracellularly in triple-negative cells or Ishikawa Cells (**Supplementary Figures 10**-**13**). These results suggest that IGFBP-6 exhibits cell cycle phase-dependent regulation in ER+ breast cancer cells, and that intracellular IGFBP-6 levels may be regulated by CDK4/6 activity.

Nocodazole treatment of MCF7 and T47D cells arrested them in G2/M and increased Cyclin B1 (**Figures 15A–C**), significantly decreased intracellular IGFBP-6, PR-A, and ER (**Figures 15B, C**), suggesting that these proteins are regulated in a cell-cycle phase-dependent manner. PR-B decreased in MCF7 cells only. In triple-negative breast cancer cells, nocodazole did not significantly affect intracellular IGFBP-6 levels. Extracellular IGFBP-6 only significantly changed in MDA-MB-231 cells, where it increased after treatment with nocodazole (**Supplementary Figure 10**).

## Discussion

In this study, a proteomic analysis identifies IGFBP-6 as a regulator of SH2D4A accumulation and cell cycle progression downstream to progesterone signaling. Knockdown of SH2D4A arrested cells in G1 and led to a reduction of cyclin B1 and p21 similarly to the knockdown of IGFBP-6. These results suggest a signaling pathway which ties progesterone, IGFBP-6, and SH2D4A to cell cycle progression in breast cancer cells. Additionally, SH2D4A levels positively correlate with IGFBP-6 in breast cancer cells and Ishikawa cells and IGFBP-6 is regulated in a cell cycle phase dependent manner downstream to CDK4/6 activation. These results mean that the protective effects of SH2D4A could stem from regulation of p21 accumulation. Decreases in the proportion of cells in G2/M could correspond to faster progression through mitosis, and SH2D4A could be protective by acting on the mitotic spindle checkpoint.

SH2D4A negatively regulates Interleukin-6 signaling by preventing STAT3 localization to the nucleus (53). Interleukin-6 is known to promote interferon signaling by increasing levels of Interferon Regulatory Factor 9 (IRF9), a regulator of interferon-stimulated gene (ISG) transcription (42, 56). IGFBP-6 knockdown increases interferon signaling, including IRF9 levels, accompanied by decreases in SH2D4A. These results could indicate that IGFBP-6 regulates IL6 signaling by increasing SH2D4A, which negatively regulates the expression of ISGs. Additionally, progesterone antagonizes interferon signaling (57, 58), suggesting that IGFBP-6 regulates inflammation in breast cancer by modulating PR and SH2D4A levels, but this requires further study.

A few of the proteins are regulated by IGFBP-6 independently of progesterone. These include ARNT, YWHAH, and ALCAM. ALCAM and YWHAH are associated with cell survival, and their expression was elevated in IGFBP-6-low cells. IGFBP-6 may be associated with suppression of these prosurvival proteins and contribute to IGFBP-6’s protective role. Low IGFBP-6 was also associated with decreases in several transcription factors, including PR, CITED4, CEBPD, and MafB. These proteins are associated with a variety of cellular processes and the consequences of which require further study. ARNT had decreased levels independently of progesterone. ARNT is a regulator of the aryl hydrocarbon receptor (Ahr), ERα, and Hypoxia-Inducible Factor-1 alpha (HIF1a) (59, 60). IGFBP-6 may also regulate hypoxia and responses to toxins, as AHR is a transcription factor that responds to xenobiotic compounds (59). These results imply that IGFBP-6 is also associated with the metabolism of xenobiotic compounds and steroid hormones.

Along with decreases in proteins associated with G2/M, there were also decreases in PR. PR has been associated with progression of the cell cycle especially as it is activated by cyclin A-CDK2 (61). Cyclin A is associated with progression through S phase (62). Treating breast cancer cells with progesterone increases IGFBP-6, which promotes exit out of G1 and accumulation of cells in G2. Inhibition of PR with RU-486 is sufficient to prevent induction of IGFBP-6 and causes cells to accumulate in G1. These results suggest that IGFBP-6 promotes exit out of G1 and regulates PR in a cell cycle phase-dependent manner. Intracellular IGFBP-6 levels are low after treatment with Abemaciclib and nocodazole whereas extracellular IGFBP-6 did not significantly decrease. These results suggest that intracellular IGFBP-6 exhibits cell cycle phase dependent expression downstream to CDK4/6. PR levels are lowest when cells are arrested in G2/M, which taken together suggest that IGFBP-6 and PR are associated with timing of the cell cycle. After progesterone activates MAPK and Cyclin D1 (17), IGFBP-6 is induced. Low IGFBP-6 arrests cells in G1 thereby decreasing PR. Future experiment needs to identify other binding partners of IGFBP-6 and the exact mechanisms regarding the timing in the levels of IGFBP-6 during cell cycle progression.

From this study, it can be concluded that IGFBP-6 regulates accumulation of SH2D4A in response to progesterone. IGFBP-6 regulates exit out of G1 and accumulation of p21 due to upstream regulation of SH2D4A. High SH2D4A is beneficial in luminal A breast cancer, suggesting IGFBP-6 is protective due to its induction of SH2D4A and p21.

## Data Availability

The datasets presented in this study can be found in online repositories. The names of the repository/repositories and accession number(s) can be found below: ftp://massive-ftp.ucsd.edu/v12/MSV000101089/, MSV000101089.

## References

[1] Smolarz B Nowak AZ Romanowicz H . Breast cancer—epidemiology, classification, pathogenesis and treatment (review of literature). Cancers. (2022) 14:2569. doi: 10.3390/cancers14102569 35626173 PMC9139759

[2] Bray F Ferlay J Soerjomataram I Siegel RL Torre LA Jemal A . Global cancer statistics 2018: GLOBOCAN estimates of incidence and mortality worldwide for 36 cancers in 185 countries. CA: A Cancer J For Clin. (2018) 68:394–424. doi: 10.3322/caac.21492 30207593

[3] Vasconcelos I Hussainzada A Berger S Fietze E Linke J Siedentopf F . The St. Gallen surrogate classification for breast cancer subtypes successfully predicts tumor presenting features, nodal involvement, recurrence patterns and disease free survival. Breast. (2016) 29:181–5. doi: 10.1016/j.breast.2016.07.016 27544822

[4] Lu H Yu X Xu Z Deng J Zhang MJ Zhang Y . Prognostic value of IGFBP6 in breast cancer: Focus on glucometabolism. Technol Cancer Res Treat. (2024) 23. doi: 10.1177/15330338241271998 39275851 PMC11402086

[5] Lariz FJ Botero PB Shoffstall I Houston KD . Insulin-like growth factor binding protein-6 modulates proliferative antagonism in response to progesterone in breast cancer. Front Endocrinol. (2024) 15. doi: 10.3389/fendo.2024.1450648 39698031 PMC11652171

[6] Wang J Luo XX Tang YL Xu JX Zeng ZG . The prognostic values of insulin-like growth factor binding protein in breast cancer. Med United States. (2019) 98. doi: 10.1097/MD.0000000000015561 31083221 PMC6531130

[7] Bach L . Insulin-like growth factor binding protein-6: The “forgotten” binding protein? Hormone Metab Res. (1999) 31:226–34. doi: 10.1055/s-2007-978723 10226806

[8] Bach LA . Current ideas on the biology of IGFBP-6: More than an IGF-II inhibitor? Growth Hormone IGF Res. (2016) 30–31:81–6. doi: 10.1016/j.ghir.2016.09.004 27681092

[9] Bach LA . Recent insights into the actions of IGFBP-6. J Cell Commun Signaling. (2015) 9:189–200. doi: 10.1007/s12079-015-0288-4 25808083 PMC4458248

[10] Baxter RC . Signaling pathways of the insulin-like growth factor binding proteins. Endocr Rev. (2023) 44:753–78. doi: 10.1210/endrev/bnad008 36974712 PMC10502586

[11] Figueroa JA Jackson JG McGuire WL Krywicki RF Yee D . Expression of insulin‐like growth factor binding proteins in human breast cancer correlates with estrogen receptor status. J Cell Biochem. (1993) 52:196–205. doi: 10.1002/jcb.240520211 7690042

[12] Shynlova O Tsui P Dorogin A Langille BL Lye SJ . Insulin-like growth factors and their binding proteins define specific phases of myometrial differentiation during pregnancy in the rat1. Biol Reprod. (2007) 76:571–8. doi: 10.1095/biolreprod.106.056929 17123939

[13] Pedroza DA Subramani R Lakshmanaswamy R . Classical and non-classical progesterone signaling in breast cancers. Cancers. (2020) 12:2440. doi: 10.3390/cancers12092440 32867363 PMC7563480

[14] Richer JK Jacobsen BM Manning NG Abel MG Horwitz KB Wolf DM . Differential gene regulation by the two progesterone receptor isoforms in human breast cancer cells. J Biol Chem. (2002) 277:5209–18. doi: 10.1074/jbc.M110090200 11717311

[15] Abdel-Hafiz H Takimoto GS Tung L Horwitz KB . The inhibitory function in human progesterone receptor N termini binds SUMO-1 protein to regulate autoinhibition and transrepression. J Biol Chem. (2002) 277:33950–6. doi: 10.1074/jbc.M204573200 12114521

[16] Groshong SD Owen GI Grimison B Schauer IE Todd MC Langan TA . Biphasic regulation of breast cancer cell growth by progesterone: Role of the cyclin-dependent kinase inhibitors, p21 and p27Kip1. Mol Endocrinol. (1997) 11:1593–607. doi: 10.1210/mend.11.11.0006 9328342

[17] Skildum A Faivre E Lange CA . Progesterone receptors induce cell cycle progression via activation of mitogen-activated protein kinases. Mol Endocrinol. (2005) 19:327–39. doi: 10.1210/me.2004-0306 15486045

[18] Lariz FJ Hernandez S Bautista-Tovar DC Houston KD . IGFBP-6 regulates breast cancer cell cycle progression by promoting exit out of G1. J Biol Chem. (2025) 302:111069. doi: 10.1016/j.jbc.2025.111069 41419198 PMC12860944

[19] Clare SE Gupta A Choi M Ranjan M Lee O Wang J . Progesterone receptor blockade in human breast cancer cells decreases cell cycle progression through G2/M by repressing G2/M genes. BMC Cancer. (2016) 16:326. doi: 10.1186/s12885-016-2355-5 27215412 PMC4878043

[20] Zheng Y Sowers JY Houston KD . IGFBP-1 expression promotes tamoxifen resistance in breast cancer cells via Erk pathway activation. Front Endocrinol. (2020) 11:233. doi: 10.3389/fendo.2020.00233 32435229 PMC7218143

[21] Posta M Győrffy B . Pathway‐level mutational signatures predict breast cancer outcomes and reveal therapeutic targets. Br J Pharmacol. (2025) 182:5734–47. doi: 10.1111/bph.70215 41057034

[22] Graw S Tang J Zafar MK Byrd AK Bolden C Peterson EC . proteiNorm – A user-friendly tool for normalization and analysis of TMT and label-free protein quantification. ACS Omega. (2020) 5:25625–33. doi: 10.1021/acsomega.0c02564 33073088 PMC7557219

[23] Huber W von Heydebreck A Sültmann H Poustka A Vingron M . Variance stabilization applied to microarray data calibration and to the quantification of differential expression. Bioinformatics. (2002) 18 Suppl 1:S96–S104. doi: 10.1093/bioinformatics/18.suppl_1.s96 12169536

[24] Thurman TJ Washam CL Alkam D Bird JT Gies A Dhusia K . proteoDA: a package for quantitative proteomics. J Open Source Software. (2023) 8:5148. doi: 10.21105/joss.05184

[25] Huber W Phipson B Wu D Hu Y Law CW Smyth GK . limma powers differential expression analyses for RNA-sequencing and microarray studies. Nucleic Acids Res. (2015) 43:e47. doi: 10.1093/nar/gkv007 25605792 PMC4402510

[26] Subramanian A Tamayo P Mootha VK Mukherjee S Ebert BL Gillette MA . Gene set enrichment analysis: A knowledge-based approach for interpreting genome-wide expression profiles. Proc Natl Acad Sci. (2005) 102:15545–50. doi: 10.1073/pnas.0506580102 16199517 PMC1239896

[27] Korotkevich G Sukhov V Budin N Shpak B Artyomov MN Sergushichev A . Fast gene set enrichment analysis. BioRxiv. (2021), 060012. doi: 10.1101/060012

[28] Milacic M Beavers D Conley P Gong C Gillespie M Griss J . The reactome pathway knowledgebase 2024. Nucleic Acids Res. (2024) 52:D672–8. doi: 10.1093/nar/gkad1025 37941124 PMC10767911

[29] Lee AJ Cai MX Thomas PE Conney AH Zhu BT . Characterization of the oxidative metabolites of 17β-estradiol and estrone formed by 15 selectively expressed human cytochrome P450 isoforms. Endocrinology. (2003) 144:3382–98. doi: 10.1210/en.2003-0192 12865317

[30] Rodriguez M Potter DA . CYP1A1 regulates breast cancer proliferation and survival. Mol Cancer Res. (2013) 11:780–92. doi: 10.1158/1541-7786.MCR-12-0675 23576571 PMC3720830

[31] Hodyl NA Stark MJ Meyer EJ Lewis JG Torpy DJ Nenke MA . High binding site occupancy of corticosteroid-binding globulin by progesterone increases fetal free cortisol concentrations. Eur J Obstet Gynecol Reprod Biol. (2020) 251:129–35. doi: 10.1016/j.ejogrb.2020.05.034 32502768

[32] Laverty HG Wakefield LM Occleston NL O’Kane S Ferguson MWJ . TGF-β3 and cancer: A review. Cytokine Growth Factor Rev. (2009) 20:305–17. doi: 10.1016/j.cytogfr.2009.07.002 19656717 PMC7294566

[33] Li J Wan X Xie D Yuan H Pei Q Luo Y . SPDEF enhances cancer stem cell-like properties and tumorigenesis through directly promoting GALNT7 transcription in luminal breast cancer. Cell Death Dis. (2023) 14:569. doi: 10.1038/s41419-023-06098-z 37633945 PMC10460425

[34] Godbole M Togar T Patel K Dharavath B Yadav N Janjuha S . Up-regulation of the kinase gene SGK1 by progesterone activates the AP-1–NDRG1 axis in both PR-positive and -negative breast cancer cells. J Biol Chem. (2018) 293:19263–76. doi: 10.1074/jbc.RA118.002894 30337371 PMC6298595

[35] Mendoza-Villanueva D Balamurugan K Ali HR Kim S-R Sharan S Johnson RC . The C/EBPδ protein is stabilized by estrogen receptor α activity, inhibits SNAI2 expression and associates with good prognosis in breast cancer. Oncogene. (2016) 35:6166–76. doi: 10.1038/onc.2016.156 27181204 PMC5112156

[36] Wesley T Berzins S Kannourakis G Ahmed N . The attributes of plakins in cancer and disease: Perspectives on ovarian cancer progression, chemoresistance and recurrence. Cell Commun Signaling. (2021) 19:55. doi: 10.1186/s12964-021-00726-x 34001250 PMC8127266

[37] Cong Y Cui Y Wang S Jiang L Cao J Zhu S . Calcium-binding protein S100P promotes tumor progression but enhances chemosensitivity in breast cancer. Front Oncol. (2020) 10:566302. doi: 10.3389/fonc.2020.566302 33042844 PMC7522638

[38] Wang Y Fan S Lu J Zhang Z Wu D Wu Z . GLUL promotes cell proliferation in breast cancer. J Cell Biochem. (2017) 118:2018–25. doi: 10.1002/jcb.25775 27791265

[39] Siraj AK Beg S Jehan Z Prabhakaran S Ahmed M R.Hussain A . ALK alteration is a frequent event in aggressive breast cancers. Breast Cancer Res. (2015) 17:127. doi: 10.1186/s13058-015-0610-3 26384210 PMC4588266

[40] Engeland K . Cell cycle arrest through indirect transcriptional repression by p53: I have a DREAM. Cell Death Diff. (2018) 25:114–32. doi: 10.1038/cdd.2017.172 29125603 PMC5729532

[41] Shabir A Qayoom H Haq BU Abo Mansoor A Abdelrahim A Ahmad I . Exploring HMMR as a therapeutic frontier in breast cancer treatment, its interaction with various cell cycle genes, and targeting its overexpression through specific inhibitors. Front Pharmacol. (2024) 15. doi: 10.3389/fphar.2024.1361424 38576486 PMC10991682

[42] Cheon H Holvey-Bates EG Schoggins JW Forster S Hertzog P Imanaka N . IFNβ-dependent increases in STAT1, STAT2, and IRF9 mediate resistance to viruses and DNA damage. EMBO J. (2013) 32:2751–63. doi: 10.1038/emboj.2013.203 24065129 PMC3801437

[43] Kim J Kang J Kang Y-L Woo J Kim Y Huh J . Ketohexokinase-A acts as a nuclear protein kinase that mediates fructose-induced metastasis in breast cancer. Nat Commun. (2020) 11:5436. doi: 10.1038/s41467-020-19263-1 33116123 PMC7595112

[44] Cochrane DR Jacobsen BM Connaghan KD Howe EN Bain DL Richer JK . Progestin regulated miRNAs that mediate progesterone receptor action in breast cancer. Mol Cell Endocrinol. (2012) 355:15–24. doi: 10.1016/j.mce.2011.12.020 22330642 PMC4716679

[45] Kulasingam V Zheng Y Soosaipillai A Leon AE Gion M Diamandis EP . Activated leukocyte cell adhesion molecule: A novel biomarker for breast cancer. Int J Cancer. (2009) 125:9–14. doi: 10.1002/ijc.24292 19322904 PMC3743675

[46] Fox SB Bragança J Turley H Campo L Han C Gatter KC . CITED4 inhibits hypoxia-activated transcription in cancer cells, and its cytoplasmic location in breast cancer is associated with elevated expression of tumor cell hypoxia-inducible factor 1α. Cancer Res. (2004) 64:6075–81. doi: 10.1158/0008-5472.CAN-04-0708 15342390

[47] Bragança J Swingler T Marques FIR Jones T Eloranta JJ Hurst HC . Human CREB-binding protein/p300-interacting transactivator with ED-rich tail (CITED) 4, a new member of the CITED family, functions as a co-activator for transcription factor AP-2. J Biol Chem. (2002) 277:8559–65. doi: 10.1074/jbc.M110850200 11744733

[48] Acun T Doberstein N Habermann JK Gemoll T Thorns C Oztas E . HLJ1 (DNAJB4) gene is a novel biomarker candidate in breast cancer. OMICS: A J Integr Biol. (2017) 21:257–65. doi: 10.1089/omi.2017.0016 28481734 PMC5586162

[49] Fang F Mo L Pan X Yang Z Huang H Zhu L . DNAJB4 promotes triple-negative breast cancer cell apoptosis via activation of the Hippo signaling pathway. Disc Oncol. (2023) 14:40. doi: 10.1007/s12672-023-00645-y 37012515 PMC10070573

[50] Mo L Liu J Yang Z Gong X Meng F Zou R . DNAJB4 identified as a potential breast cancer marker: Evidence from bioinformatics analysis and basic experiments. Gland Surg. (2020) 9:1955–72. doi: 10.21037/gs-20-431 33447546 PMC7804547

[51] Lu Y Chang P Bian J Zhu L . Dnali1 is a prognosis-related biomarker and correlates with immune infiltrates in low grade glioma. Cancer Biomarkers. (2023) 38:393–407. doi: 10.3233/CBM-230139 37955080 PMC12412861

[52] Li T Li W Lu J Liu H Li Y Zhao Y . Sh2d4a regulates cell proliferation via the ERα/PLC-γ/PKC pathway. BMB Rep. (2009) 42:516–22. doi: 10.5483/BMBRep.2009.42.8.516 19712589

[53] Ploeger C Waldburger N Fraas A Goeppert B Pusch S Breuhahn K . Chromosome 8p tumor suppressor genes SH2D4A and SORBS3 cooperate to inhibit interleukin‐6 signaling in hepatocellular carcinoma. Hepatology. (2016) 64:828–42. doi: 10.1002/hep.28684 27311882 PMC5098049

[54] Yuki R Ikeda Y Yasutake R Saito Y Nakayama Y . Sh2d4a promotes centrosome maturation to support spindle microtubule formation and mitotic progression. Sci Rep. (2023) 13:2067. doi: 10.1038/s41598-023-29362-w 36739326 PMC9899277

[55] Faivre EJ Daniel AR Hillard CJ Lange CA . Progesterone receptor rapid signaling mediates serine 345 phosphorylation and tethering to specificity protein 1 transcription factors. Mol Endocrinol. (2008) 22:823–37. doi: 10.1210/me.2007-0437 18202149 PMC2276470

[56] Erb HHH Langlechner R Moser PL Handle F Casneuf T Verstraeten K . Il6 sensitizes prostate cancer to the antiproliferative effect of IFNα2 through IRF9. Endocrine-Related Cancer. (2013) 20:677–89. doi: 10.1530/ERC-13-0222 23913484 PMC3753051

[57] Goodman ML Trinca GM Walter KR Papachristou EK D’Santos CS Li T . Progesterone receptor attenuates STAT1-mediated IFN signaling in breast cancer. J Immunol. (2019) 202:3076–86. doi: 10.4049/jimmunol.1801152 30936295 PMC6504603

[58] Walter KR Balko JM Hagan CR . Progesterone receptor promotes degradation of STAT2 to inhibit the interferon response in breast cancer. OncoImmunology. (2020) 9. doi: 10.1080/2162402X.2020.1758547 32391191 PMC7199813

[59] Swedenborg E Pongratz I . Ahr and ARNT modulate ER signaling. Toxicology. (2010) 268:132–8. doi: 10.1016/j.tox.2009.09.007 19778576

[60] Wood SM Gleadle JM Pugh CW Hankinson O Ratcliffe PJ . The role of the aryl hydrocarbon receptor nuclear translocator (ARNT) in hypoxic induction of gene expression. J Biol Chem. (1996) 271:15117–23. doi: 10.1074/jbc.271.25.15117 8662957

[61] Narayanan R Adigun AA Edwards DP Weigel NL . Cyclin-dependent kinase activity is required for progesterone receptor function: Novel role for cyclin A/Cdk2 as a progesterone receptor coactivator. Mol Cell Biol. (2005) 25:264–77. doi: 10.1128/MCB.25.1.264-277.2005 15601848 PMC538783

[62] Matthews HK Bertoli C de Bruin RAM . Cell cycle control in cancer. Nat Rev Mol Cell Biol. (2022) 23:74–88. doi: 10.1038/s41580-021-00404-3 34508254

