# Supplementary material for: IGFBP-6 regulates SH2D4A expression to promote breast cancer cell cycle progression in response to progesterone
Source: Front Endocrinol (Lausanne). 2026 Jun 30;17:1856096. doi: 10.3389/fendo.2026.1856096 (PMC13364574; doi:10.3389/fendo.2026.1856096)
Supplement: Supplementary file 1 [file Presentation1.pptx]

## Slide 1
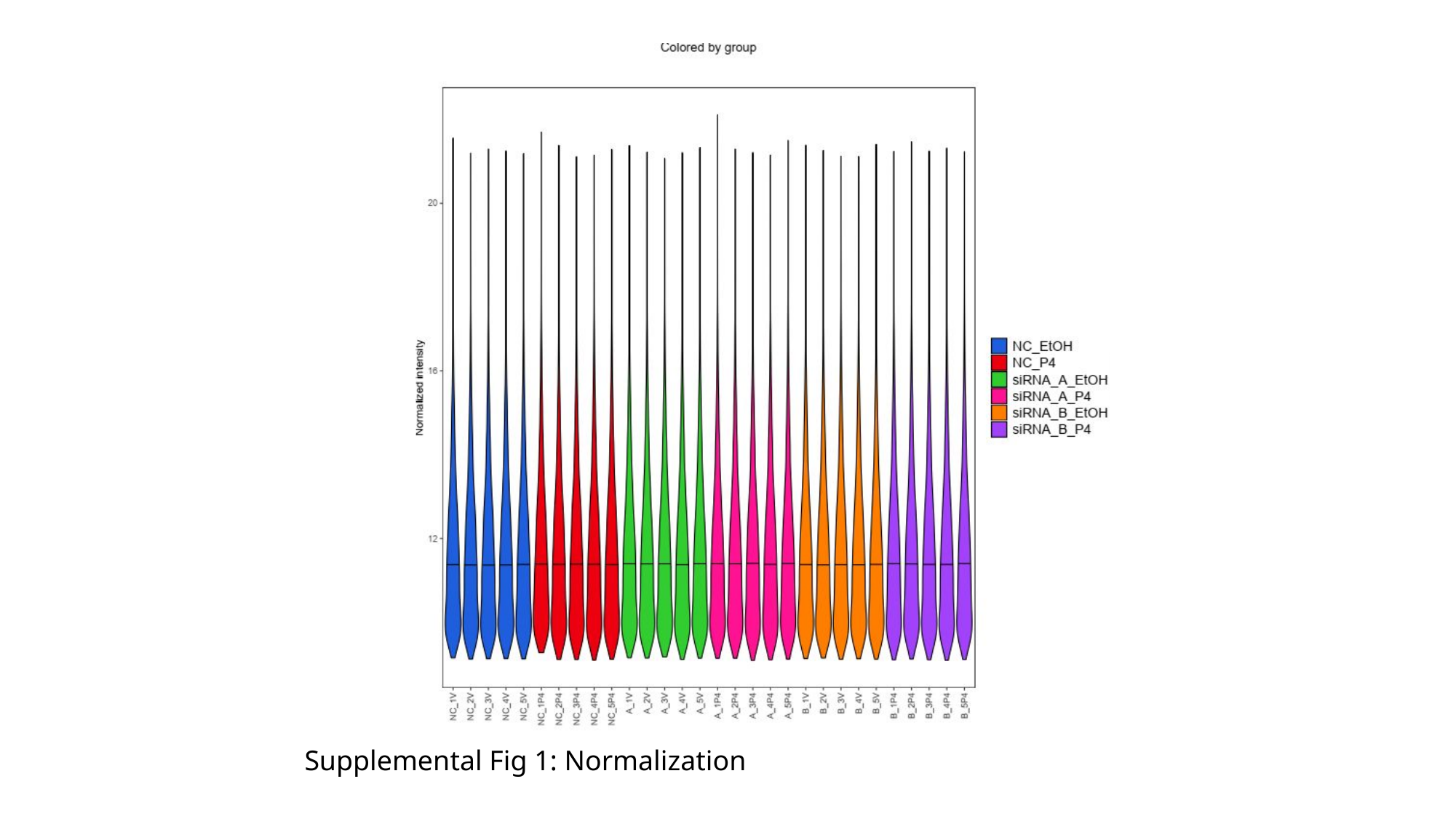

Supplemental Fig 1: Normalization

## Slide 2
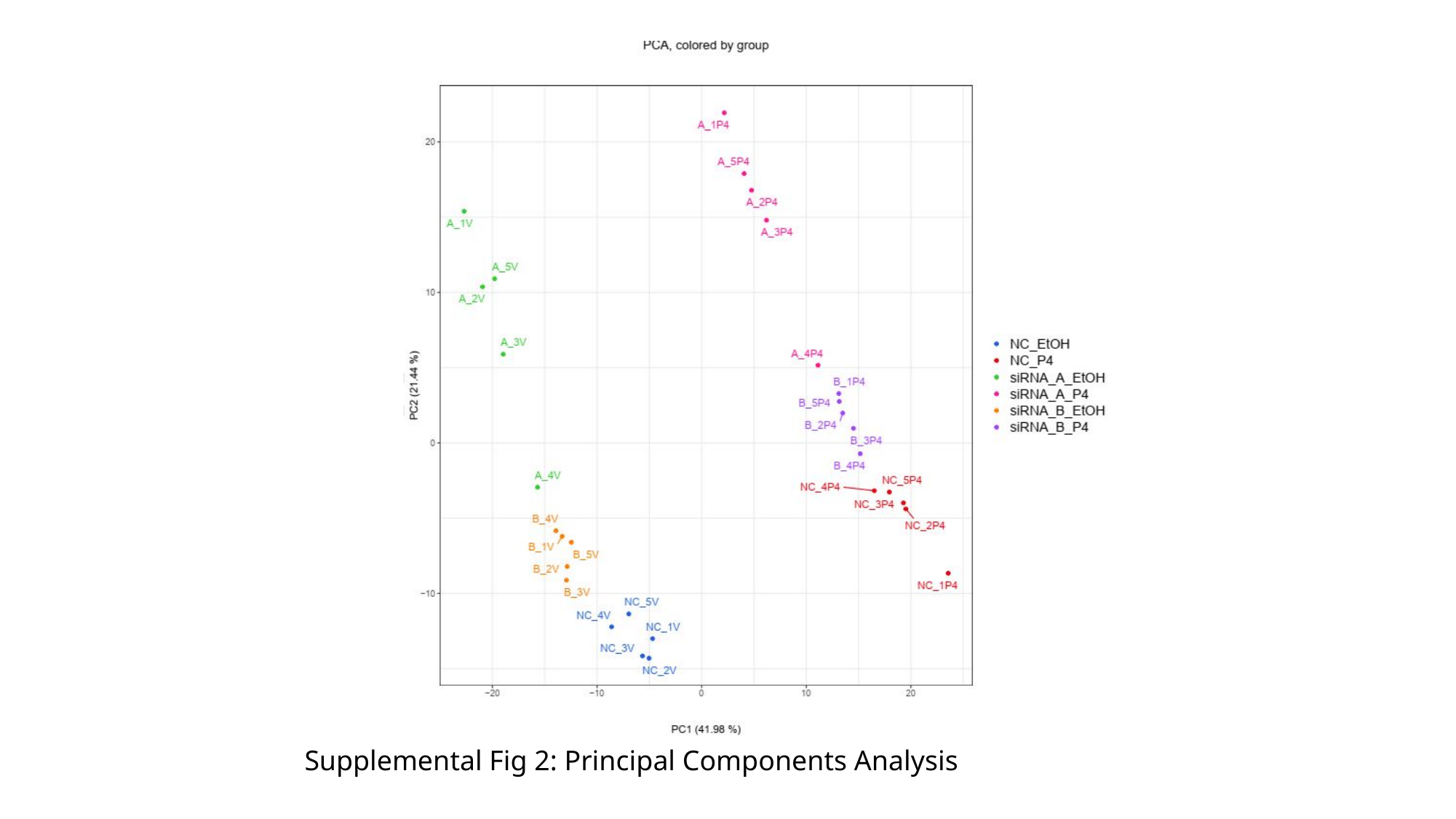

Supplemental Fig 2: Principal Components Analysis

## Slide 3
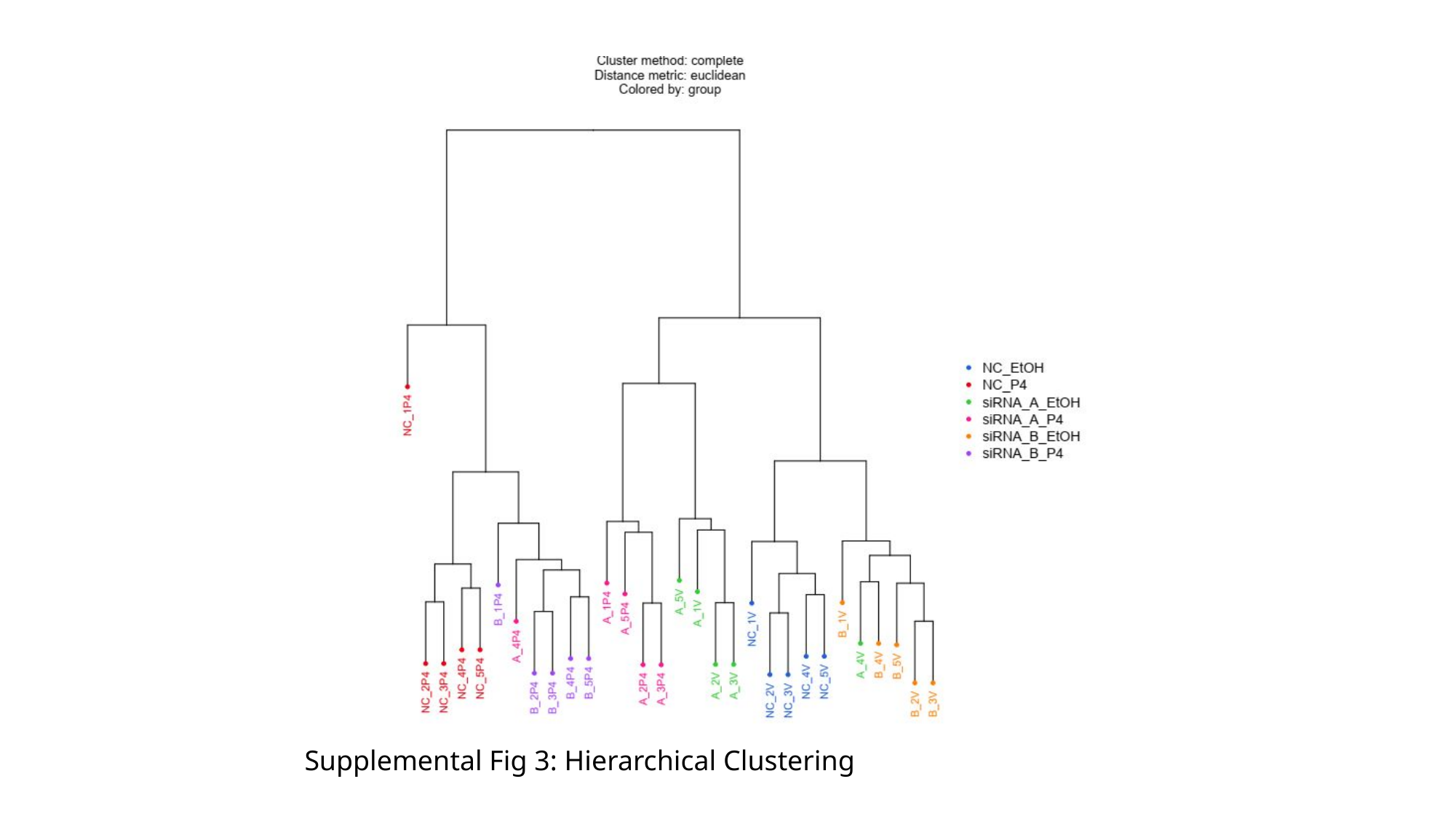

Supplemental Fig 3: Hierarchical Clustering

## Slide 4
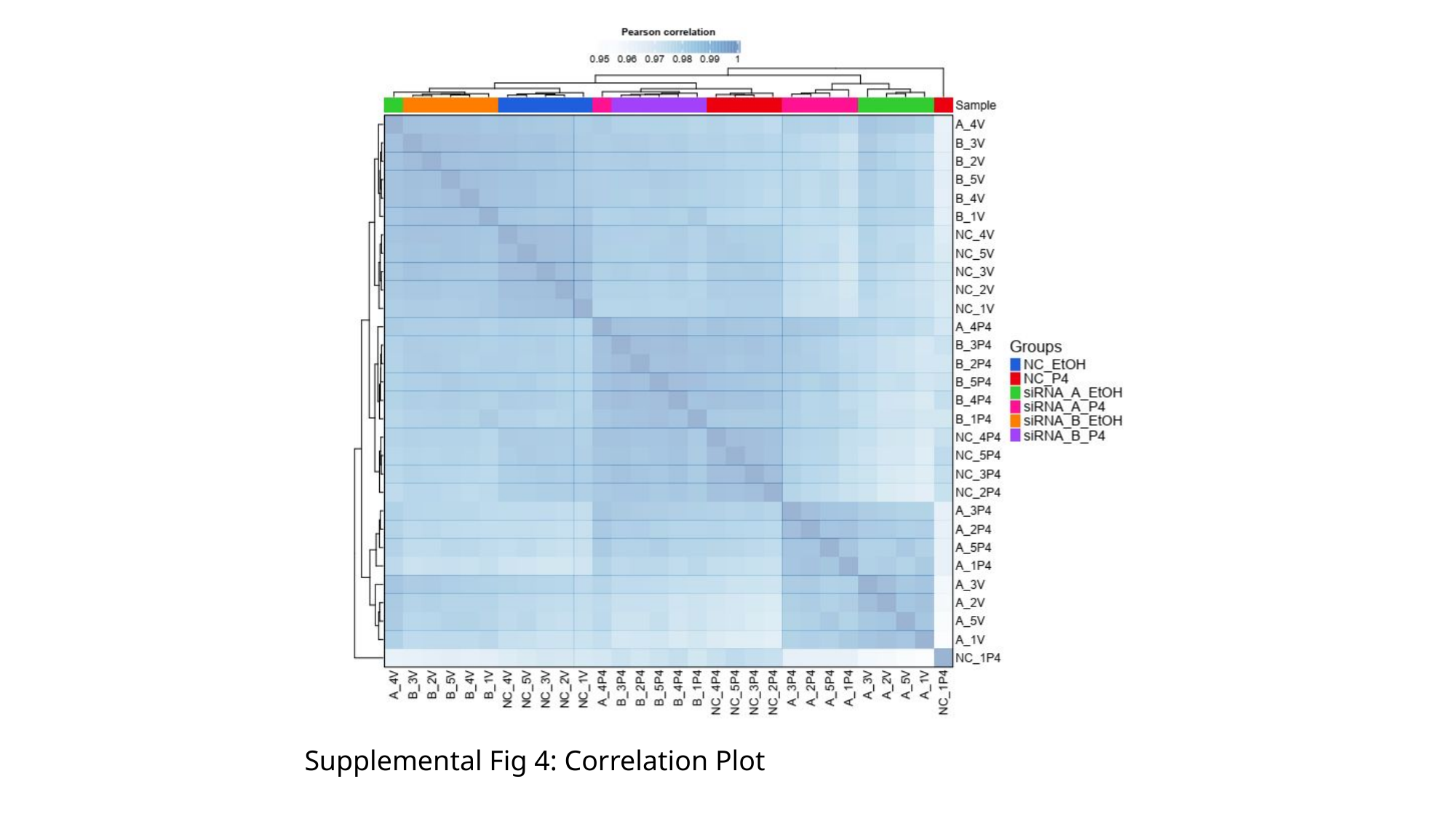

Supplemental Fig 4: Correlation Plot

## Slide 5
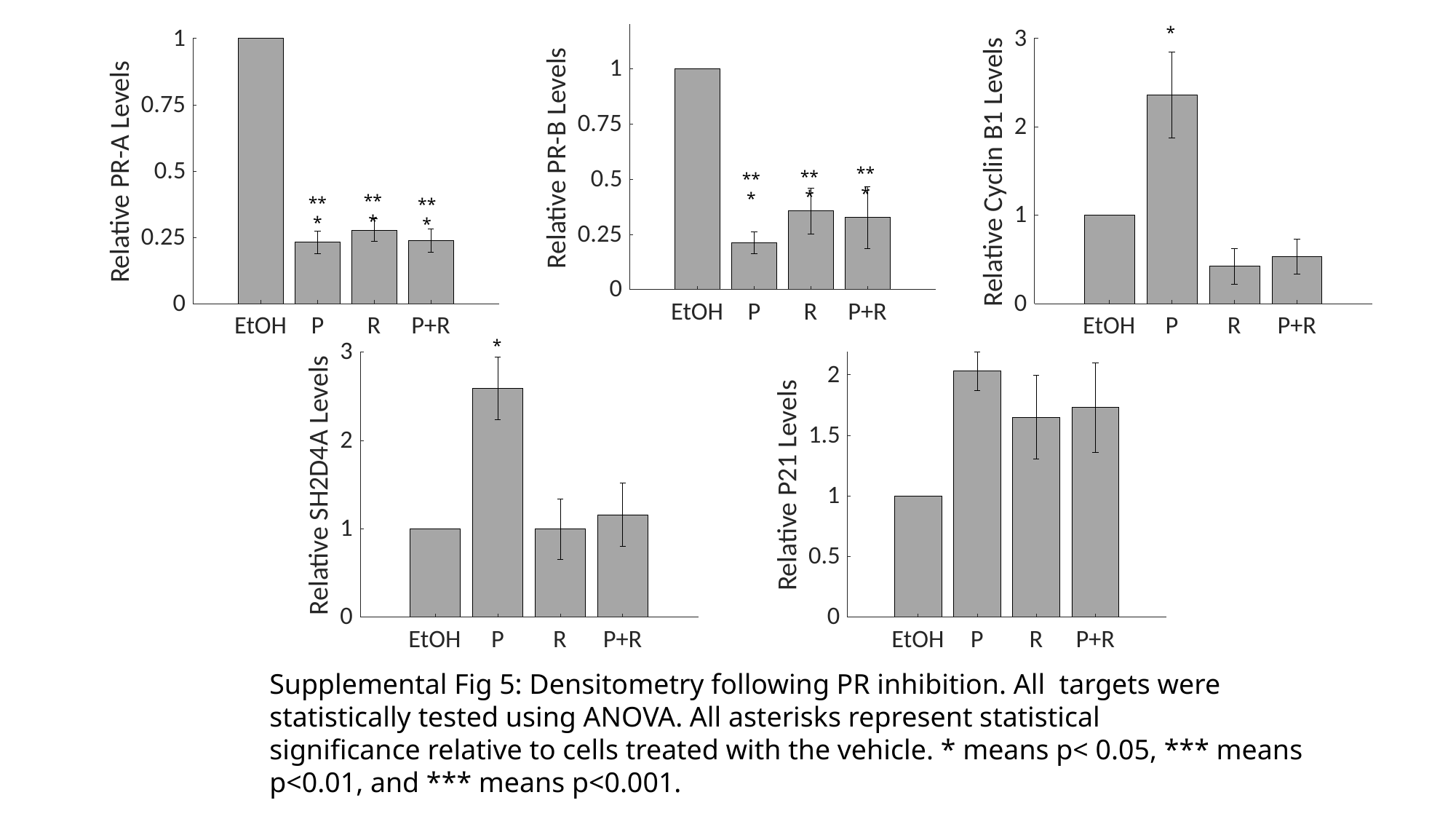

*
***
***
***
***
***
***
*
Supplemental Fig 5: Densitometry following PR inhibition. All targets were statistically tested using ANOVA. All asterisks represent statistical significance relative to cells treated with the vehicle. * means p< 0.05, *** means p<0.01, and *** means p<0.001.

## Slide 6
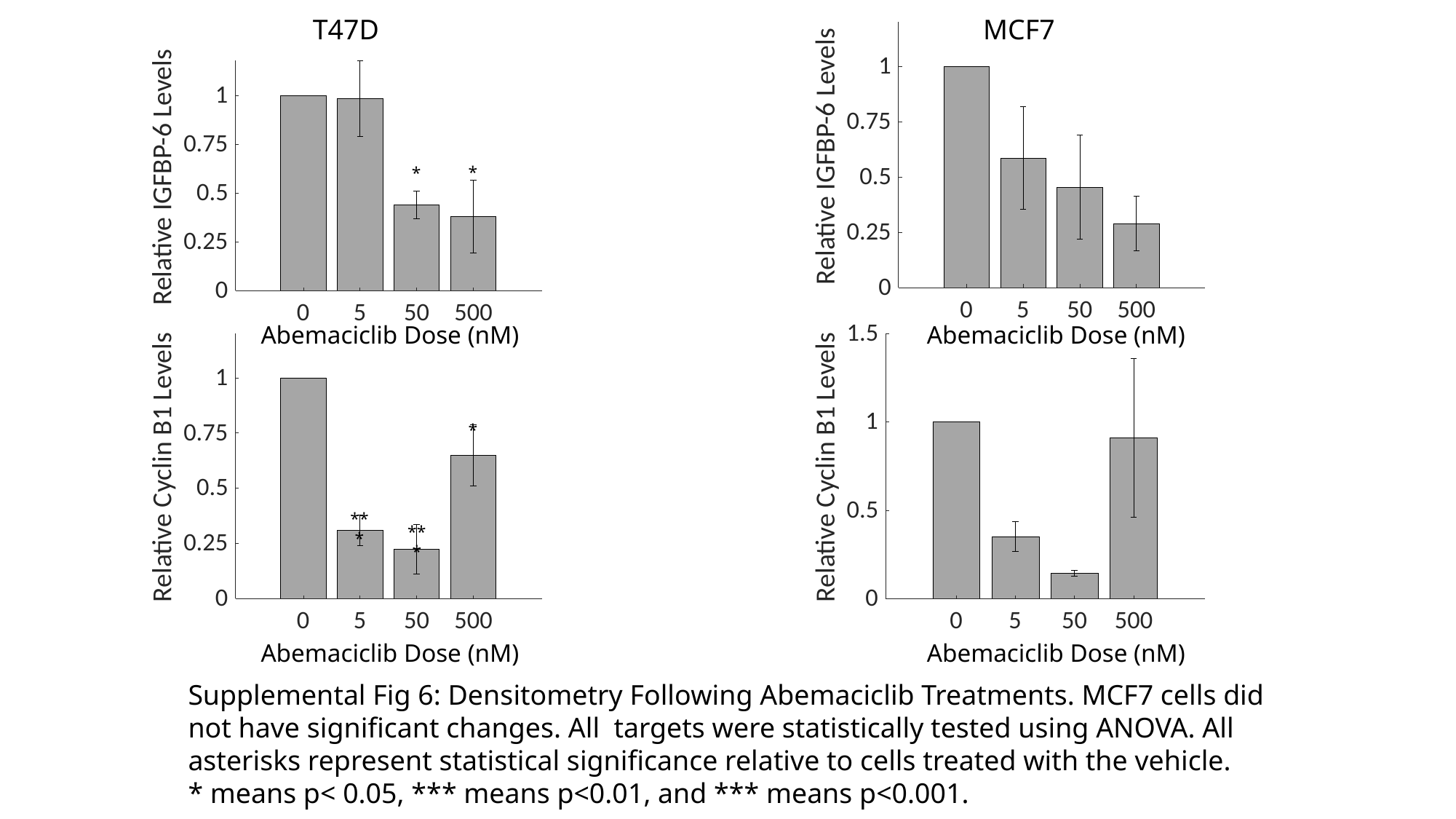

T47D
MCF7
*
*
Abemaciclib Dose (nM)
Abemaciclib Dose (nM)
*
***
***
Abemaciclib Dose (nM)
Abemaciclib Dose (nM)
Supplemental Fig 6: Densitometry Following Abemaciclib Treatments. MCF7 cells did not have significant changes. All targets were statistically tested using ANOVA. All asterisks represent statistical significance relative to cells treated with the vehicle. * means p< 0.05, *** means p<0.01, and *** means p<0.001.

## Slide 7
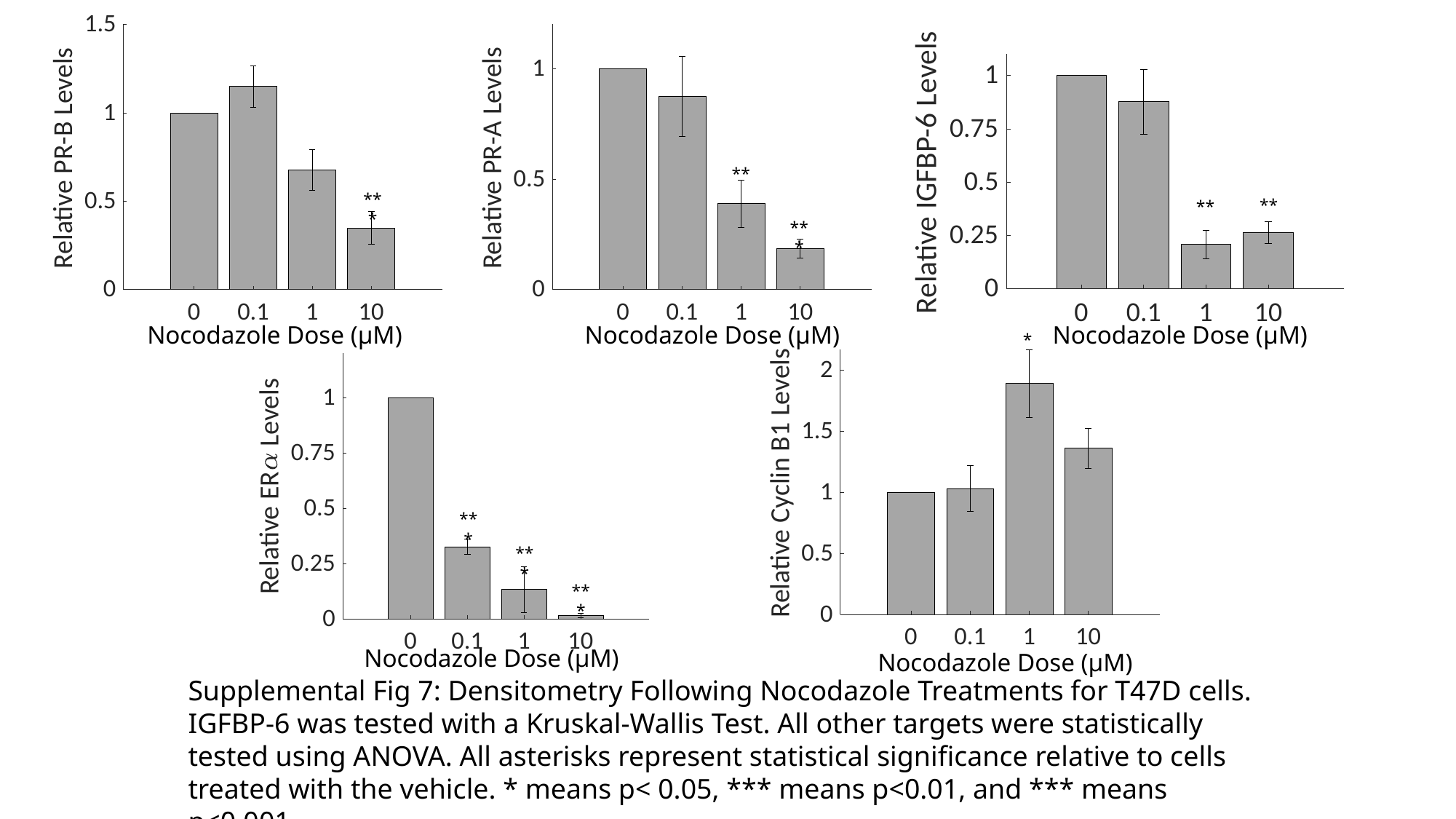

**
***
**
**
***
Nocodazole Dose (μM)
Nocodazole Dose (μM)
Nocodazole Dose (μM)
*
***
***
***
Nocodazole Dose (μM)
Nocodazole Dose (μM)
Supplemental Fig 7: Densitometry Following Nocodazole Treatments for T47D cells. IGFBP-6 was tested with a Kruskal-Wallis Test. All other targets were statistically tested using ANOVA. All asterisks represent statistical significance relative to cells treated with the vehicle. * means p< 0.05, *** means p<0.01, and *** means p<0.001.

## Slide 8
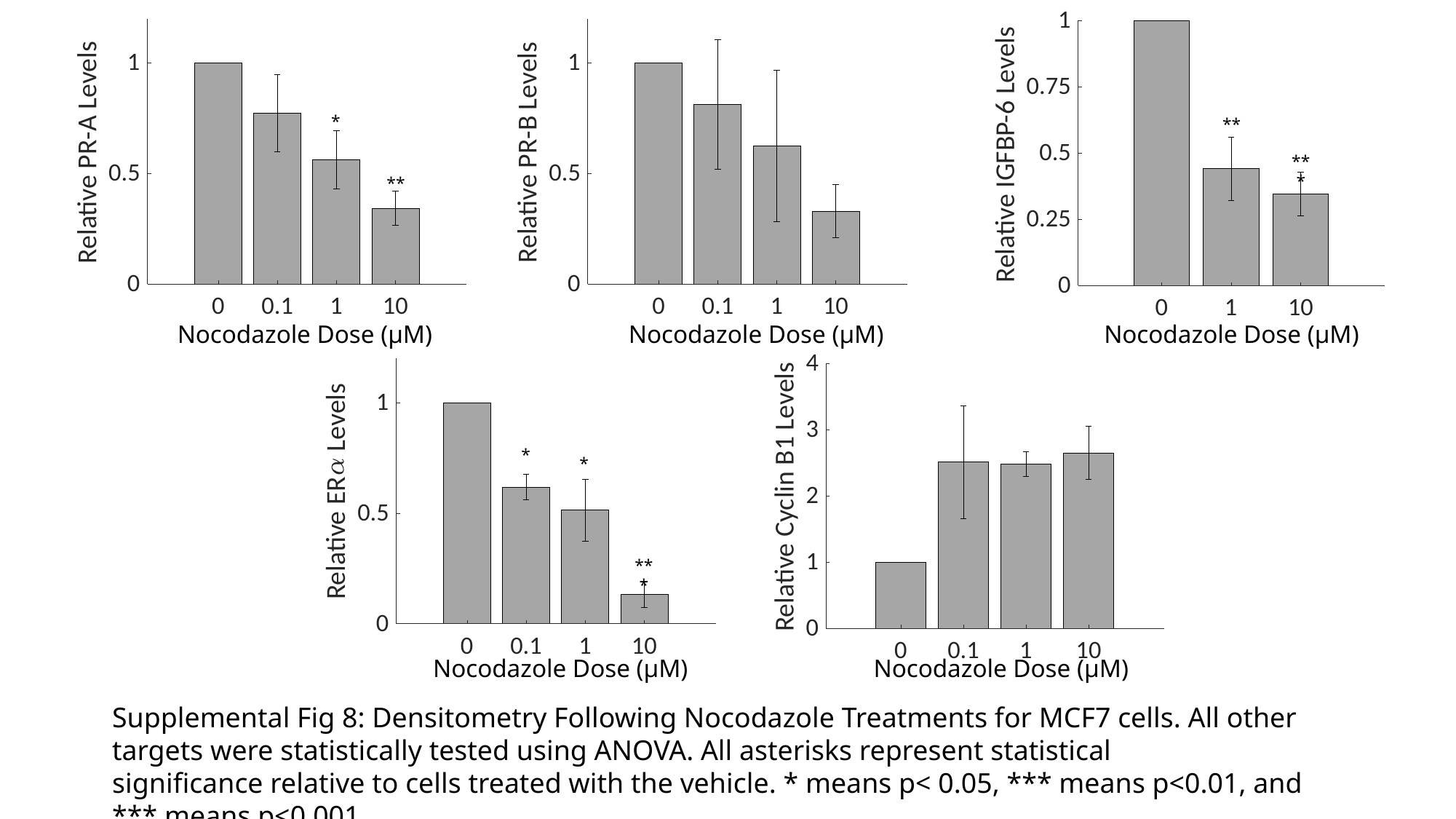

*
**
***
**
Nocodazole Dose (μM)
Nocodazole Dose (μM)
Nocodazole Dose (μM)
*
*
***
Nocodazole Dose (μM)
Nocodazole Dose (μM)
Supplemental Fig 8: Densitometry Following Nocodazole Treatments for MCF7 cells. All other targets were statistically tested using ANOVA. All asterisks represent statistical significance relative to cells treated with the vehicle. * means p< 0.05, *** means p<0.01, and *** means p<0.001.

## Slide 9
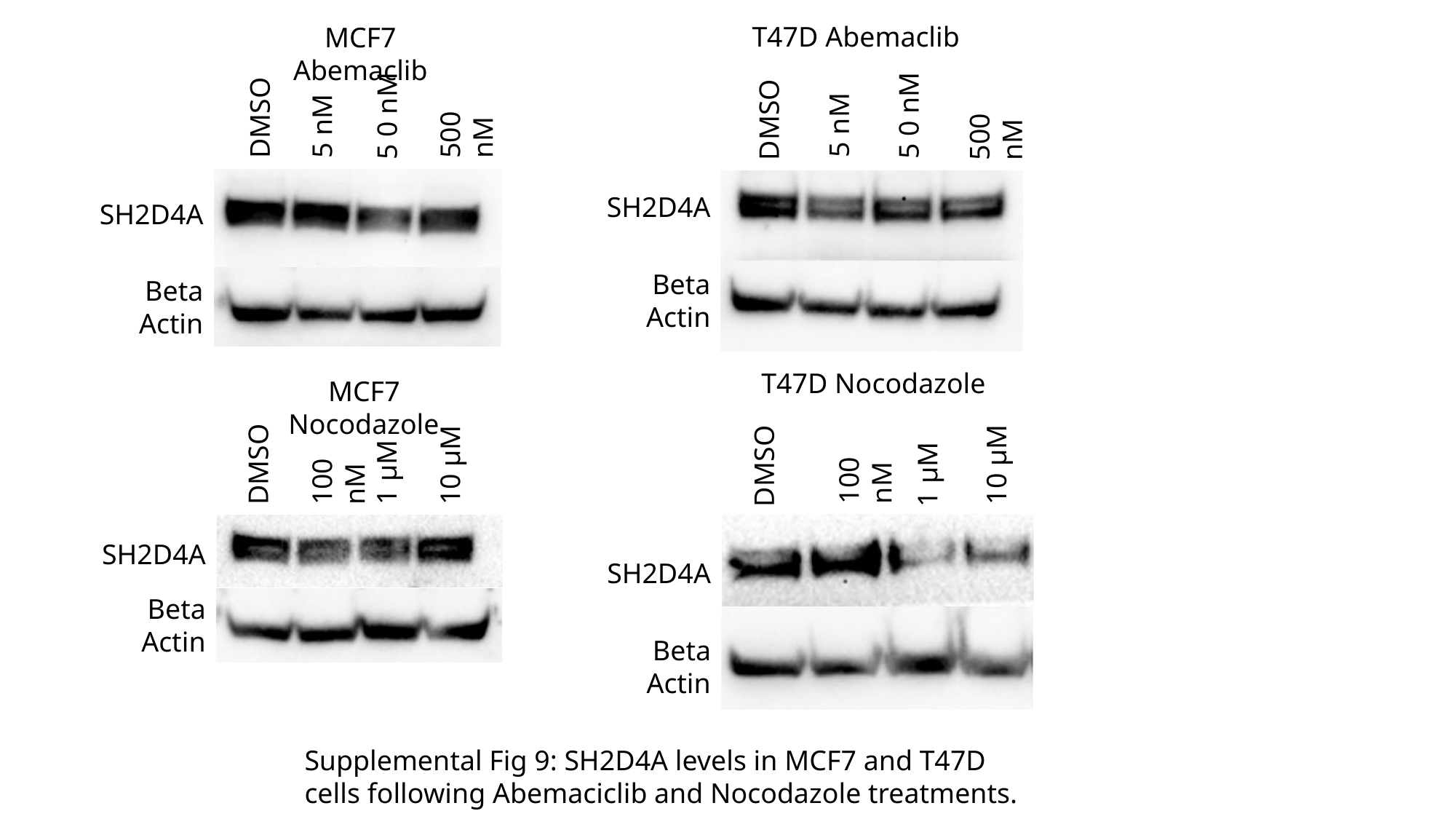

T47D Abemaclib
MCF7 Abemaclib
5 nM
5 nM
DMSO
500 nM
5 0 nM
5 0 nM
DMSO
500 nM
SH2D4A
SH2D4A
Beta Actin
Beta Actin
T47D Nocodazole
MCF7 Nocodazole
100 nM
1 μM
10 μM
DMSO
100 nM
10 μM
DMSO
1 μM
SH2D4A
SH2D4A
Beta Actin
Beta Actin
Supplemental Fig 9: SH2D4A levels in MCF7 and T47D cells following Abemaciclib and Nocodazole treatments.

## Slide 10
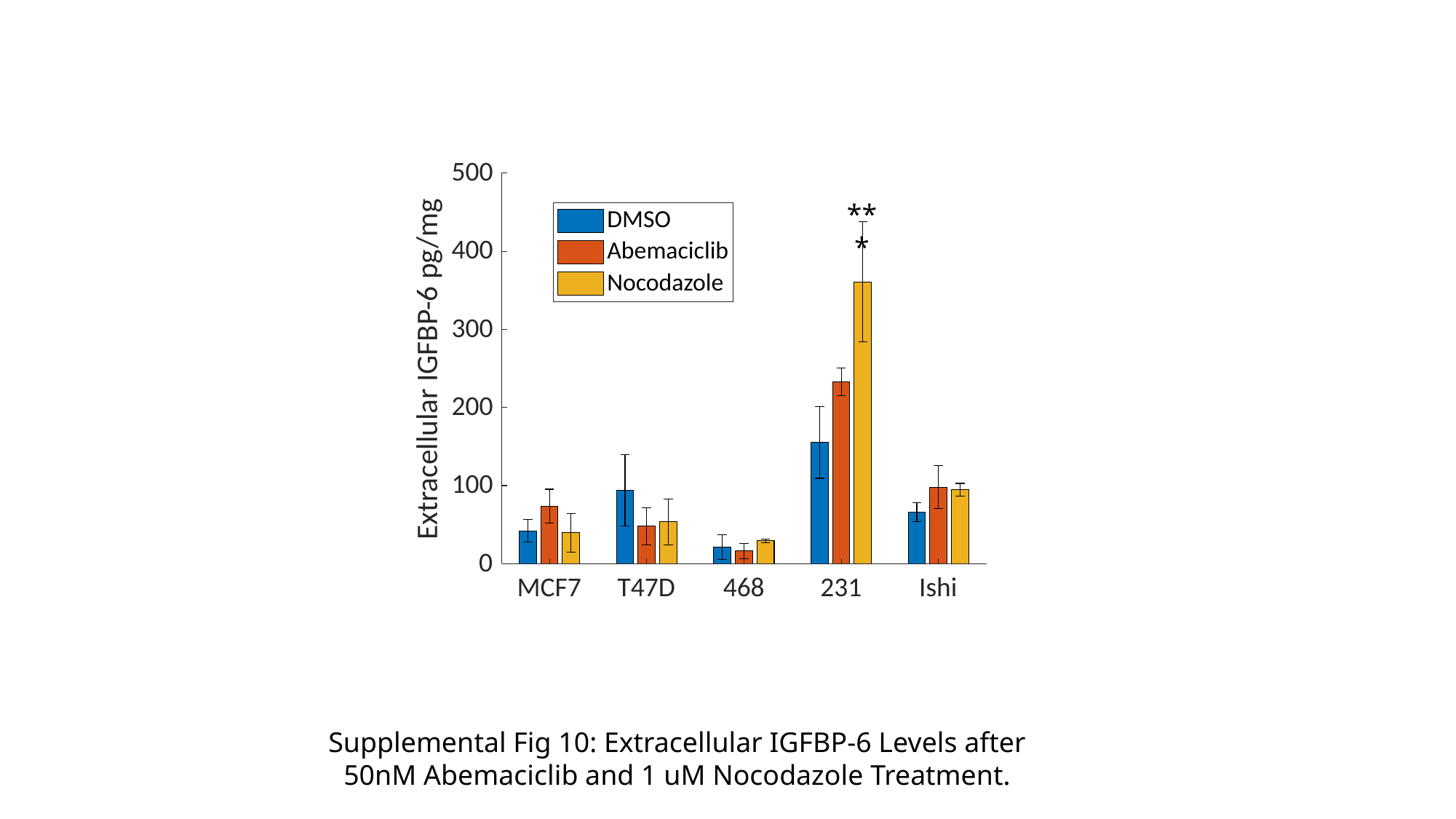

***
Supplemental Fig 10: Extracellular IGFBP-6 Levels after 50nM Abemaciclib and 1 uM Nocodazole Treatment.

## Slide 11
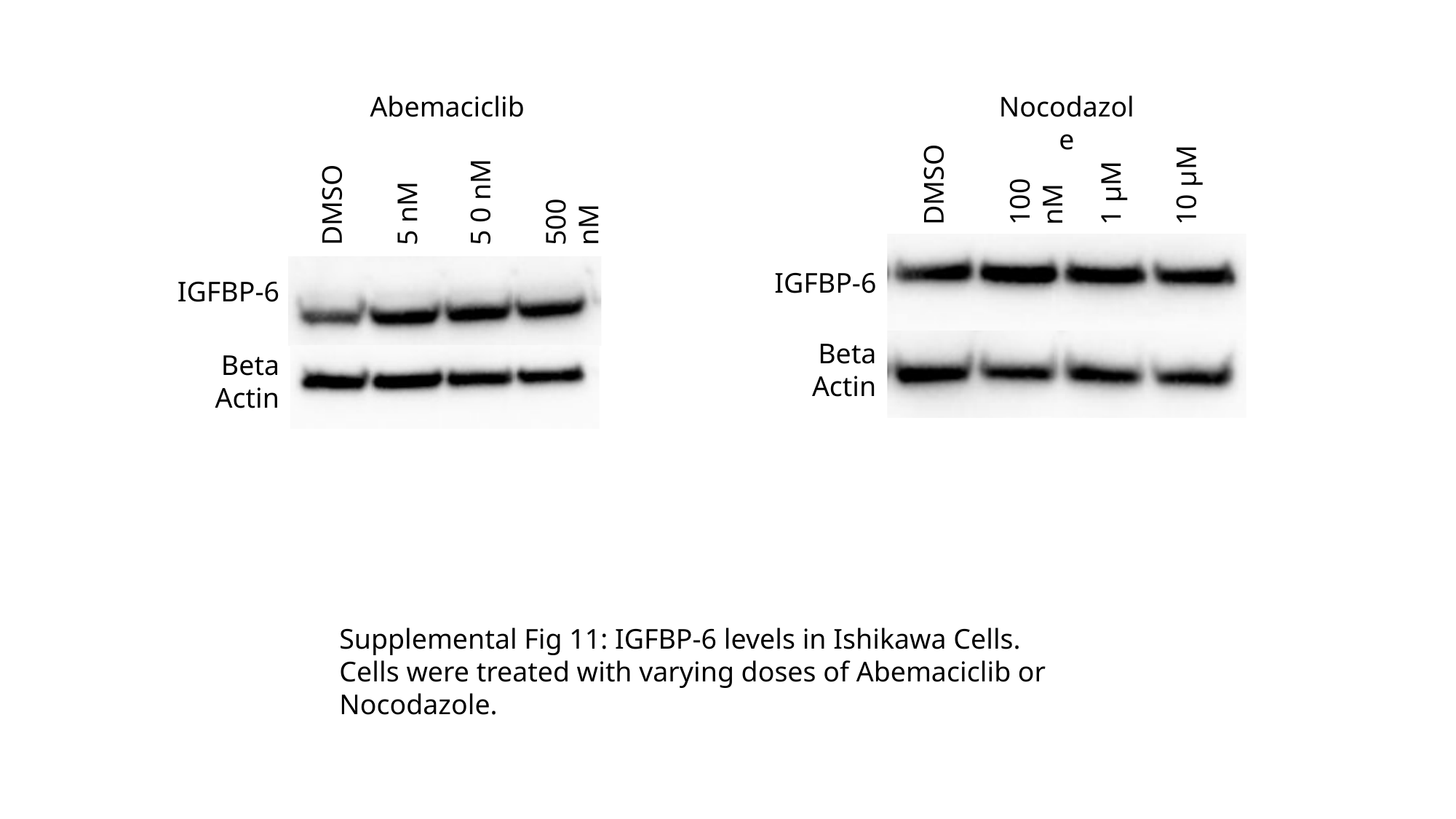

Abemaciclib
Nocodazole
100 nM
1 μM
10 μM
DMSO
5 0 nM
DMSO
5 nM
500 nM
IGFBP-6
IGFBP-6
Beta Actin
Beta Actin
Supplemental Fig 11: IGFBP-6 levels in Ishikawa Cells. Cells were treated with varying doses of Abemaciclib or Nocodazole.

## Slide 12
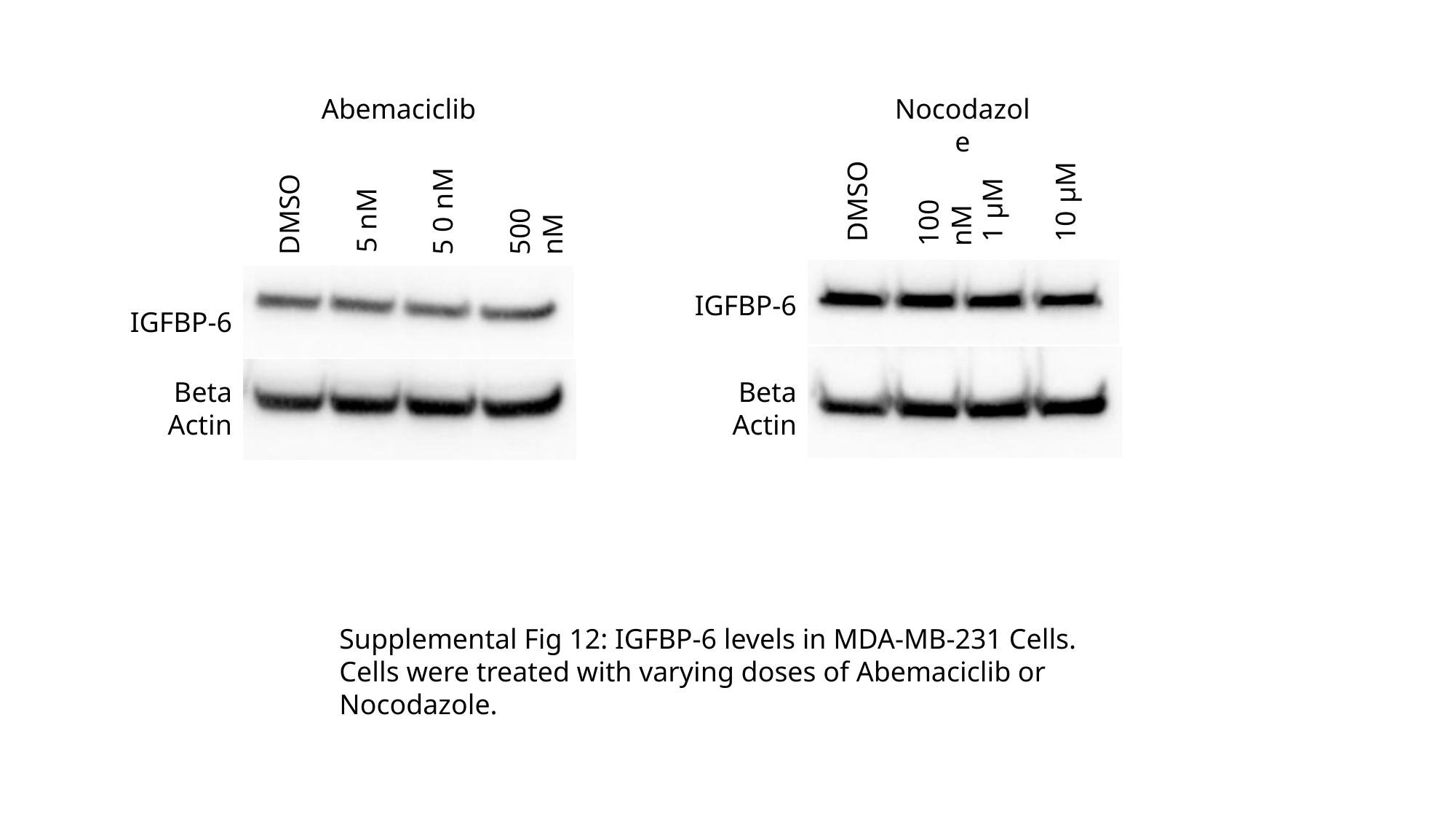

Abemaciclib
Nocodazole
DMSO
1 μM
10 μM
100 nM
5 nM
DMSO
5 0 nM
500 nM
IGFBP-6
IGFBP-6
Beta Actin
Beta Actin
Supplemental Fig 12: IGFBP-6 levels in MDA-MB-231 Cells. Cells were treated with varying doses of Abemaciclib or Nocodazole.

## Slide 13
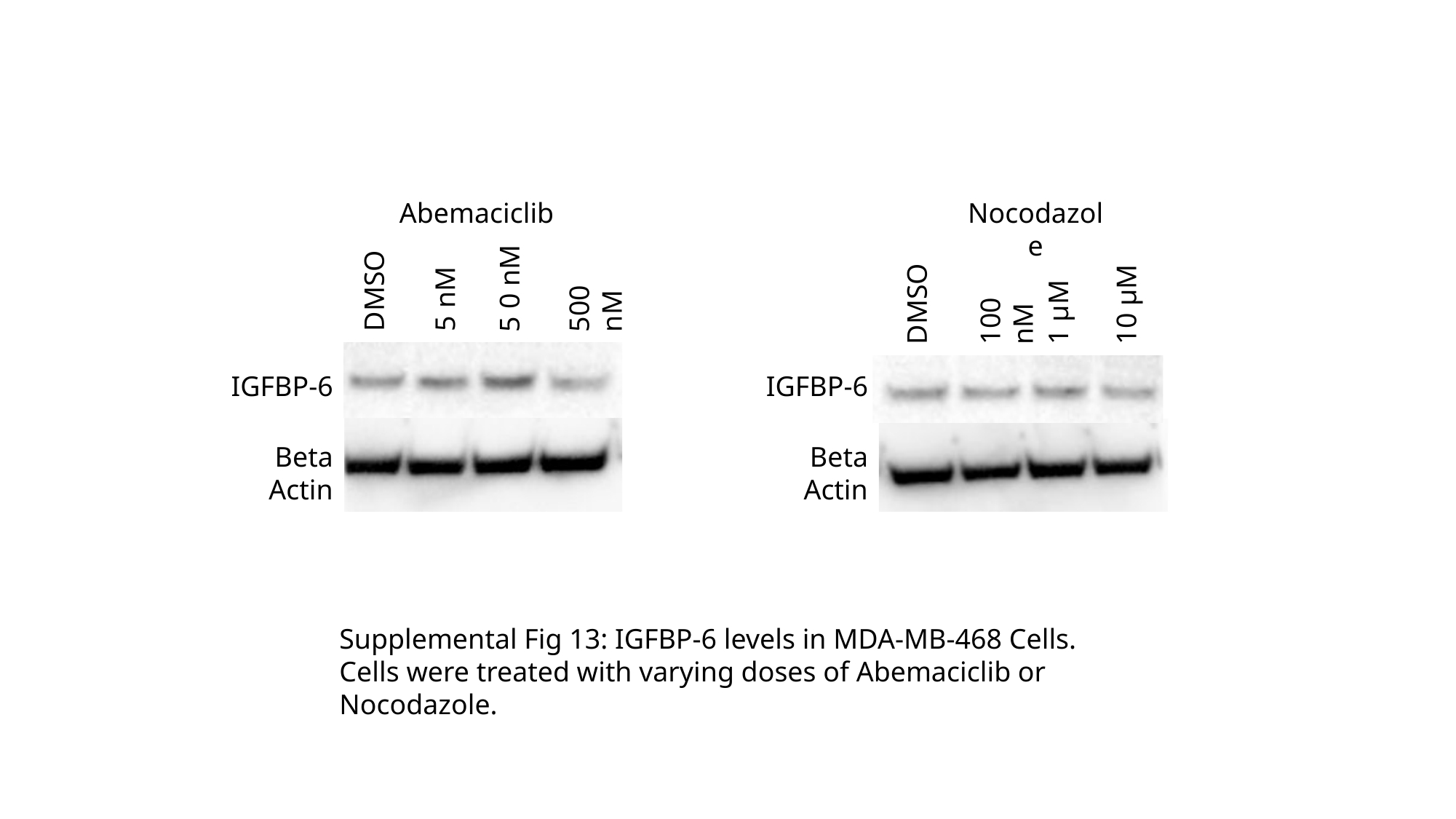

Abemaciclib
Nocodazole
DMSO
5 nM
5 0 nM
500 nM
DMSO
100 nM
1 μM
10 μM
IGFBP-6
IGFBP-6
Beta Actin
Beta Actin
Supplemental Fig 13: IGFBP-6 levels in MDA-MB-468 Cells. Cells were treated with varying doses of Abemaciclib or Nocodazole.
